# Glomerulonephritis and autoimmune vasculitis are independent of P2RX7 but may depend on alternative inflammasome pathways

**DOI:** 10.1002/path.5890

**Published:** 2022-05-02

**Authors:** Maria Prendecki, Stephen P McAdoo, Tabitha Turner‐Stokes, Ana Garcia‐Diaz, Isabel Orriss, Kevin J Woollard, Jacques Behmoaras, H Terence Cook, Robert Unwin, Charles D Pusey, Timothy J Aitman, Frederick WK Tam

**Affiliations:** ^1^ Centre for Inflammatory Disease, Department of Immunology and Inflammation Imperial College London, Hammersmith Campus London UK; ^2^ Department of Comparative Biomedical Sciences Royal Veterinary College London UK; ^3^ Programme in Cardiovascular and Metabolic Disorders and Centre for Computational Biology, Duke‐NUS Medical School Singapore Singapore; ^4^ Department of Renal Medicine, Division of Medicine University College London London UK; ^5^ Centre for Genomic & Experimental Medicine Institute of Genetics and Molecular Medicine, University of Edinburgh Edinburgh UK; ^6^ Present address: Early Clinical Development, Research and Early Development, Cardiovascular, Renal and Metabolism (CVRM), BioPharmaceuticals R&D, AstraZeneca Cambridge UK; ^7^ Present address: Bioscience Renal, Research and Early Development, Cardiovascular, Renal and Metabolism (CVRM), BioPharmaceuticals R&D, AstraZeneca Cambridge UK

**Keywords:** P2RX7, inflammasome, NLRP3, IL‐1β, glomerulonephritis, vasculitis, caspase‐1, autoimmunity, inflammation

## Abstract

P2RX7, an ionotropic receptor for extracellular adenosine triphosphate (ATP), is expressed on immune cells, including macrophages, monocytes, and dendritic cells and is upregulated on nonimmune cells following injury. P2RX7 plays a role in many biological processes, including production of proinflammatory cytokines such as interleukin (IL)‐1β via the canonical inflammasome pathway. P2RX7 has been shown to be important in inflammation and fibrosis and may also play a role in autoimmunity. We have developed and phenotyped a novel P2RX7 knockout (KO) inbred rat strain and, taking advantage of the human‐resembling unique histopathological features of rat models of glomerulonephritis, we induced three models of disease: nephrotoxic nephritis, experimental autoimmune glomerulonephritis, and experimental autoimmune vasculitis. We found that deletion of P2RX7 does not protect rats from models of experimental glomerulonephritis or the development of autoimmunity. Notably, treatment with A‐438079, a P2RX7 antagonist, was equally protective in WKY WT and P2RX7 KO rats, revealing its ‘off‐target’ properties. We identified a novel ATP/P2RX7/K^+^ efflux‐independent and caspase‐1/8‐dependent pathway for the production of IL‐1β in rat dendritic cells, which was absent in macrophages. Taken together, these results comprehensively establish that inflammation and autoimmunity in glomerulonephritis is independent of P2RX7 and reveals the off‐target properties of drugs previously known as selective P2RX7 antagonists. Rat mononuclear phagocytes may be able to utilise an ‘alternative inflammasome’ pathway to produce IL‐1β independently of P2RX7, which may account for the susceptibility of P2RX7 KO rats to inflammation and autoimmunity in glomerulonephritis. © 2022 The Authors. *The Journal of Pathology* published by John Wiley & Sons Ltd on behalf of The Pathological Society of Great Britain and Ireland.

## Introduction

The P2X7 receptor (P2RX7), an ionotropic receptor for extracellular adenosine triphosphate (ATP), is expressed on immune cells including macrophages, monocytes, and dendritic cells [1, 2, 3]. A key P2RX7 function is production of the proinflammatory cytokine interleukin (IL)‐1β as part of a ‘two‐signal’ model involving canonical inflammasome activation. Cell priming and accumulation of pro‐IL‐1β in the cytoplasm is triggered by stimulation of toll‐like receptors (TLR) signalling via nuclear factor kappa B (NFκB) [4]. A second signal, such as P2RX7 activation by ATP, leads to K^+^ efflux from the cell, assembly of the NLRP3 inflammasome, activation of caspase‐1, and subsequent cleavage of IL‐1β to its mature form [5]. Active caspase‐1 also cleaves gasdermin‐D (GSDMD), leading to GSDMD pores in the plasma membrane, mature IL‐1β release, and pyroptosis [6, 7]. Studies using P2RX7 knockout (KO) mice and P2RX7 antagonists have shown that IL‐1β production is dependent on this two‐step model in murine macrophages. Recently, several different P2RX7‐dependent and ‐independent mechanisms in human monocytes have been suggested, whereby secretion of active IL‐1β occurs in response to stimulation with lipopolysaccharide (LPS) alone, without a second signal [8, 9, 10, 11, 12, 13, 14, 15, 16]. Canonical, noncanonical, and alternative inflammasome activation pathways have all been implicated, with several potential mechanisms suggested, including autocrine release of ATP, maxiK channels, caspase‐11 (or caspase‐4/5 in humans), and caspase‐8 (supplementary material, Figure S1).

Several studies suggest the involvement of P2RX7 and IL‐1β in autoimmune and inflammatory diseases, including glomerulonephritis (GN), in both rodents and humans [2, 17]. In nephrotoxic nephritis (NTN) in Wistar Kyoto (WKY) rats there is increased glomerular P2RX7 and IL‐1β gene expression, coinciding with the onset of proteinuria at day 4 [2]. WKY rats are uniquely susceptible to NTN compared to Lewis (LEW) rats, and even under basal conditions, macrophages from WKY rats express more P2RX7 and NLRP3 than LEW macrophages; this difference may underlie, in part, their increased susceptibility [18]. In NTN in mice, infiltrating glomerular leucocytes produce IL‐1β, and both IL‐1β‐deficient and P2RX7‐deficient mice have decreased severity of GN [19, 20, 21]. In rats, both IL‐1 and P2RX7 antagonism are effective treatments for NTN, suggesting a role for this axis in disease pathogenesis [21, 22]. In contrast to IL‐1, a role for NLRP3 and caspase‐1 in the pathogenesis of GN is more controversial, and may differ depending on the phase and mechanism of glomerular injury. In the heterologous (early) phase of NTN in mice, there was no change in disease severity in mice deficient for NLRP3, ASC, or caspase‐1 [23]. In contrast, in the autologous (later) phase of murine NTN, there is increased glomerular expression of NLRP3, ASC, and IL‐1β mRNA with detection of mature IL‐1β protein, and a deficiency of NLRP3 and ASC results in improved renal function and glomerular injury [24].

P2RX7 may also play a role in the development of autoimmunity; treatment with a P2RX7 antagonist decreased the severity of experimental autoimmune encephalomyelitis (EAE) in mice [25]. However, studies of EAE in two different P2RX7 KO mice generated contrasting results, with one strain of mice developing increased disease severity and one strain protected from disease [26, 27].

In this study we developed and phenotyped a novel P2RX7 KO rat on a Wistar‐Kyoto (WKY) background and explored the hypothesis that P2RX7 plays a role in experimental models of GN and autoimmunity using three different models of glomerulonephritis and vasculitis. We used two distinct allosteric modulators of P2RX7, described to be selective, and which have been shown to be effective in other rodent models of inflammatory disease [21, 28, 29, 30, 31]. We investigated dependence on P2RX7 for IL‐1β production by different mononuclear phagocytes.

## Materials and methods

See Supplementary materials and methods for additional details.

### Study approval

All animal procedures were carried out in accordance with the regulations of the UK Animals (Scientific Procedures) Act (1986). Animal experiments were carried out under UK Home Office Project Licenses.

### Animal husbandry

Homozygous P2RX7 KO rats were bred in‐house, and WKY WT were purchased from Charles River (Saffron Walden, UK).

### Development of a novel WKY‐P2RX7 KO rat strain

A global P2RX7 KO on a WKY background was created at Imperial College London using zinc finger nuclease (ZFN) technology to generate a 2 base pair insertion in exon 10 of *P2rx7*.

### Animal models

NTN was induced in male rats aged 8–10 weeks by 0.1 ml of nephrotoxic serum (rabbit antirat GBM antiserum) given intravenously [32]. Experimental autoimmune glomerulonephritis (EAG) was induced by immunizing 6–8‐week‐old female rats with 100 μg α3(IV)NC1 in complete Freund's adjuvant (CFA) [33]. Experimental autoimmune vasculitis (EAV) was induced by immunising 6–8‐week‐old female rats, immunised with purified 1,600 μg/kg human myeloperoxidase (MPO; Calbiochem, San Diego, CA, USA) in CFA with additional *Mycobacterium butyricum*. Animals also received 500 ng of pertussis toxin intraperitoneally on day 0 and day 2 [34].

### Antagonist studies

A‐438079 (Sai Life Sciences, Hyderabad, India) 275 μmol/kg was administered twice daily by intraperitoneal injection. AZ11657312 (Astra Zeneca, Cambridge, UK) 60 mg/kg was administered twice daily by oral gavage. Control animals received an equivalent administration of vehicle.

### Assessment of renal and lung injury

Haematuria was quantified by dipstick analysis, and proteinuria by the sulphosalicylic acid method [35]. To assess glomerular injury, 50 consecutive glomeruli in kidney sections were assessed for crescent formation and expressed as the mean proportion for each animal. Immunohistochemistry was performed on formalin‐fixed paraffin‐embedded tissues using the primary antibodies antirat CD68 (1:500, ED1, Bio‐Rad, Hercules, CA, USA), antirat CD43 (1:100, W3/13, Bio‐Rad), or antirat MHC II (1:100, OX6, Bio‐Rad) and the number of positive cells per glomerular cross‐section quantified using automated image analysis. Lung haemorrhage was graded by visual inspection at the time of cull, and by quantification of hemosiderin‐containing cells by microscopic assessment using automated image analysis.

### Assessment of autoantibody response

Circulating α3(IV)NC1 or MPO antibodies were assayed in serum by direct enzyme‐linked immunoassay (ELISA) created in‐house using microplates coated with α3(IV)NC1 or MPO and pooled sera from historic experiments as a standard curve. The presence of deposited rat IgG and rabbit IgG was assessed using FITC‐labelled antibodies for direct immunofluorescence of frozen kidney sections.

### B‐cell ELIspot

MPO‐reactive B‐cells were identified in splenocytes from rats with EAV using ELIspot. ELIspot plates were coated with hMPO and splenocytes incubated for 48 h. Spots per well were quantified using an ELISpot plate reader and software (ELISpot 4.0, Autoimmun Diagnostika, Strassberg, Germany).

### Culture of nephritic glomeruli *ex vivo*

Glomeruli were isolated from animals with NTN, EAG, and controls by differential sieving of whole‐kidney tissue as previously described [36]. Glomeruli were cultured *ex vivo* for 48 h at 37 °C in 5% CO_2_ and cytokine levels in supernatants measured using ELISAs.

### Western blotting

Cell and tissue lysates, and acetone‐precipitated protein from supernatant were resolved by sodium dodecylsulphate–polyacrylamide gel electrophoresis, transferred to nitrocellulose membranes, and probed with the following primary antibodies: GAPDH (1:1,000, AF5718, R&D Systems, Minneapolis, MN, USA), P2RX7 004 (1:200, APR004, Alomone, Jerusalem, Israel), IL‐1β (1:1,000, AF501, R&D), IL‐18 (R&D), Caspase‐1 (1:1,000, ab179515 Abcam, Cambridge, UK) and Caspase‐8 (1:1,000, D35G2, Cell Signaling Technology, Danvers, MA, USA).

### RT‐PCR

RNA extracted from cells and tissues was reverse‐transcribed into cDNA using an iScript cDNA synthesis kit (Bio‐Rad). qPCR was performed using qPCRBIO SyGreen mix (PCR Biosystems, London, UK). Fold‐changes were calculated using the 2^−ΔΔCT^ method relative to *Pgk1*. Endpoint reverse transcribed polymerase chain reaction (RT‐PCR) was carried out using goTaq green mastermix (Promega, Madison, WI, USA). Agarose gel electrophoresis of PCR products was performed and the QIAquick PCR purification kit (Qiagen, Hilden, Germany) was used to clean up PCR products for sequencing analysis. The primers used are listed in Supplementary materials and methods and supplementary material, Table S1.

### Micro‐computed X‐ray tomographic (μCT) analysis

The tibiae and femora were isolated from 6‐ and 12‐week‐old P2RX7 KO and WKY WT rats and used for μCT analysis of trabecular and cortical bone parameters (SkyScan 1172). Analysis of isolated bones was performed without knowledge of the age or genotype. Images were reconstructed, analysed, and visualised using SkyScan NRecon, CTAn, and CTVol software (Bruker, Billerica, MA, USA).

### Primary cell isolation

Bone marrow‐derived macrophages (BMDM) and bone marrow‐derived dendritic cells (BMDC) were differentiated from rat bone marrow cells using rat MCSF (Peprotech, Rocky Hill, NJ, USA), and rat GM‐CSF (Peprotech) and IL‐4 (Peprotech), respectively. Monocytes were isolated from rat whole blood using cell sorting using a BD FACSAria II flow cytometer. Monocytes were gated as Lin−/CD45+/CD172a+ and CD43/His48 used to generate a monocyte ‘waterfall’ [37]. Classical and nonclassical monocytes were pooled for stimulation experiments.

### Stimulation of BMDM and BMDC

Cells were stimulated with 1 μg/ml LPS (Invivogen, San Diego, CA, USA) followed by ATP (Sigma, St. Louis, MO, USA) 5 mm. Selected cultures also contained 10 U/ml apyrase (Sigma). For inhibitor experiments, cells were preincubated with vehicle, A‐438079 (Tocris, Bristol, UK), AZ 11657312 (AstraZeneca), Ac‐YVAD‐cmk (Invivogen), or Z‐IETD‐FMK (InvivoGen, Toulouse, France). For experiments in the presence of excess potassium, KCl was added to media to raise K^+^ levels to the indicated concentration. Secreted IL‐1β in supernatant was measured using a commercially available sandwich ELISA kit (R&D) according to the manufacturer's instructions. Western blotting was carried out as described above.

### YO‐PRO‐1 uptake

To assess P2RX7 pore formation and YO‐PRO‐1 uptake, YO‐PRO‐1 Iodide (491/509) (Invitrogen, ThermoFisher Scientific, Waltham, MA, USA) was added to cell suspensions 5 min prior to analysis. Baseline fluorescence was measured for 1 min, 150 μm BzATP added, and dye uptake recorded over 5 min on a BD Accuri C6 flow cytometer (BD Biosciences, Franklin Lakes, NJ, USA).

### ATP luciferase assay

For quantitative analysis of ATP in supernatants the Molecular Probes ATP determination kit was used (Molecular Probes, Eugene, OR, USA) and samples read using a FLUOstar Omega plate reader (BioTek, Winooski, VT, USA).

### Statistical analyses

Statistical analysis was conducted using GraphPad Prism 9.0 (GraphPad Software, San Diego, CA, USA). All data are reported as median per group with interquartile range unless otherwise stated. Comparison between groups used the Mann–Whitney *U* test, or, for multiple variables, the Kruskal–Wallis test with Dunn's *post hoc* test.

## Results

### Development of a novel P2RX7 KO WKY rat strain

**Figure 1:**
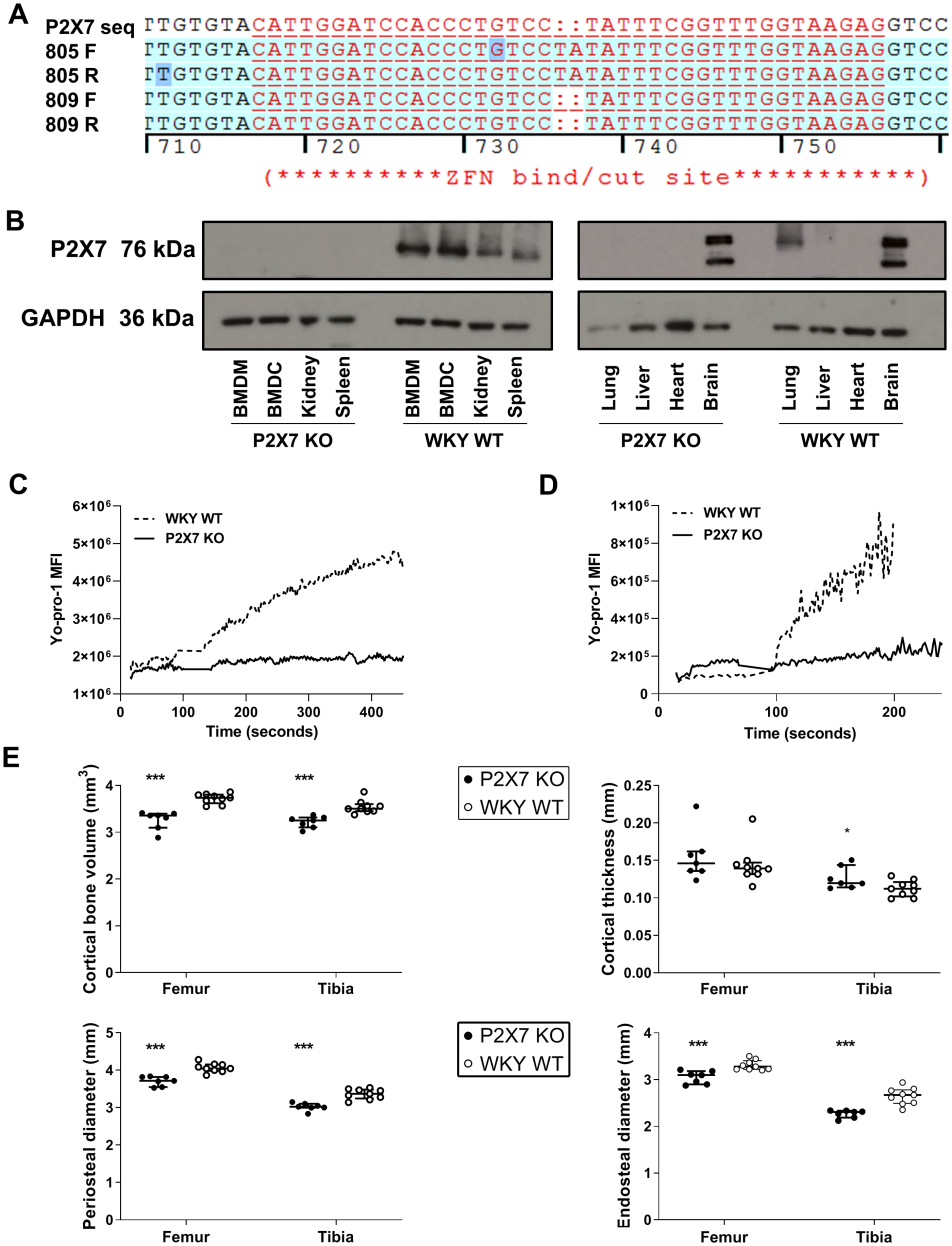
Characterisation of a novel P2RX7 knockout rat. (A) Electropherogram of the P2RX7 gene sequences with ZFN cut site and the 2 bp insertion (ringed) in pup 805, and in another WT pup 809 without the 2 bp insertion. (B) Representative western blot of tissue homogenate and cell lysates from P2RX7 KO and WKY WT rats using an antibody directed against the C‐terminus of P2RX7. This is representative of ≥3 biological replicates for each tissue and cell type. (C) YO‐PRO‐1 fluorescent dye uptake by BMDM and (D) YO‐PRO‐1 fluorescent dye uptake by BMDC. 150 μm BzATP was added to cells at the arrows. Images are representative of three biological replicates for each cell type. (E) Bone phenotyping data from μCT imaging of age and sex matched P2RX7 KO and WKY WT rat femur and tibia at 12 weeks. See also supplementary material, Figures S2 and S3. BMDM, Bone marrow‐derived macrophages. BMDC, bone marrow‐derived dendritic cells. Data are shown as median with IQR and a Mann–Whitney test was used to compare P2RX7 KO to WKY WT within each bone type. ***p < 0.001.

A KO rat strain was developed using ZFN technology targeting exon 10 of P2RX7, resulting in a two‐base pair (2 bp) insertion at the target site. (Figure 1A, and supplementary material, Figure S2A). Sequencing of genomic DNA and of RT‐PCR products/cDNA of cells and tissues from P2RX7 KO rats from the homozygous breeding colony confirmed the 2 bp insertion was present, and that rats are a global KO without chimaerism.

P2RX7 protein was not detected by western blotting in tissue and cell lysates from the P2RX7 KO, except in brain tissue, where a double band was observed in both WT and P2RX7 KO (Figure 1B). Sequencing of RT‐PCR products from brain tissue of P2RX7 KO rats identified that the 2 bp insertion was present, suggesting this may represent a non‐P2RX7 protein. P2RX7 expression in rodent brain is debated, and has been addressed by others—it is unclear if this band represents a P2RX7‐related protein or an as a yet‐unrecognised brain‐specific P2RX7 splice variant [38, 39]. Bone marrow‐derived cells were unable to take up YO‐PRO‐1 fluorescent dye following stimulation with BzATP, confirming loss of functional P2RX7 receptors (Figure 1C,D).

P2RX7 KO animals were noted to be smaller than age‐ and sex‐matched WKY WT controls and μCT imaging revealed a relatively small bone phenotype, in keeping with the known role for P2RX7 in bone homeostasis [40, 41]. At 12 weeks of age, P2RX7 KO rats had 14% and 10% lower cortical bone volume of the femur (*p* < 0.0001) and tibia (*p* = 0.0002), respectively, compared to age‐ and sex‐matched WKY WT controls (Figure 1E). All trabecular bone parameters were unaffected, and young rats (6 weeks) displayed no skeletal changes (supplementary material, Figure S2B–D). P2RX7 KO rats did not display any renal phenotype, with no evidence of urinary abnormalities, histological abnormalities, or derangement of renal function in rats up to 24 weeks of age (supplementary material, Figure S3).

### P2RX7 KO does not protect from renal or lung injury in rat GN or vasculitis

We investigated the role of P2RX7 in three rat models of GN: (1) NTN, a passive model of crescentic GN induced by immunisation with heterologous rabbit antirat glomerular basement membrane (GBM) serum [32]; (2) EAG, an autoimmune model of anti‐GBM disease induced by immunisation with recombinant rat α3(IV)NC1, the Goodpasture autoantigen [42]; and (3) EAV, an autoimmune model of antimyeloperoxidase (MPO) vasculitis induced by immunisation with human MPO [43]. We identified upregulation of *P2rx7*, *Nlrp3*, and *Il1b* mRNA in renal cortical tissue from WKY WT rats with GN in all three models, suggesting a role for P2RX7 in disease pathogenesis. The greatest increase was in EAG; this model having the greatest degree of renal inflammation at the timepoints studied (supplementary material, Figure S4A–E). Assessment of protein expression identified upregulation of P2RX7 and caspase‐1, again with the greatest increase in EAG (supplementary material, Figure S4F–I).

**Figure 2:**
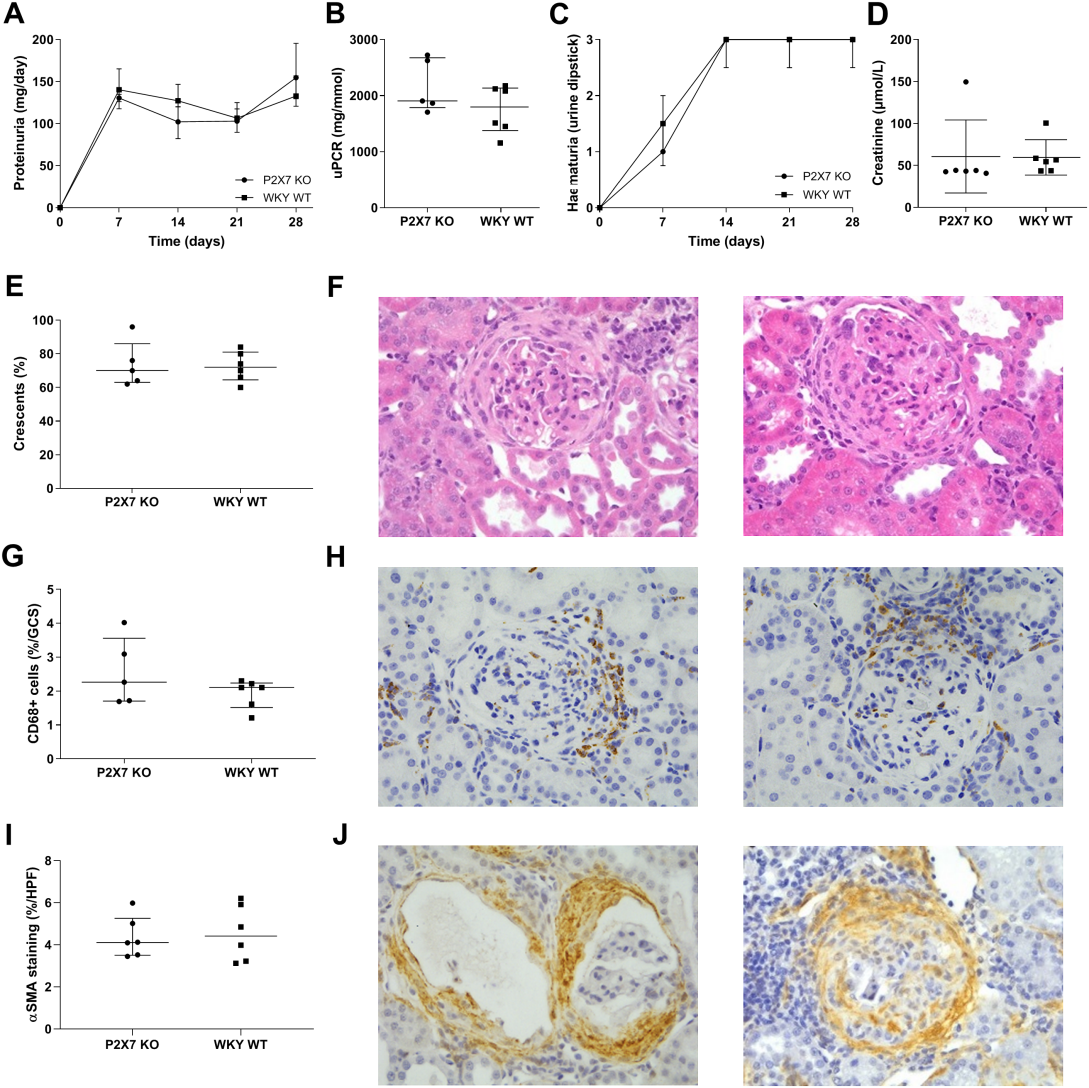
P2RX7 KO rats are not protected from nephrotoxic nephritis. (A) Proteinuria during the 28‐day course of NTN. (B) Urine protein: creatinine ratio (uPCR) at day 28 after induction of NTN. (C) Haematuria during the 28‐day course of NTN. (D) Excretory renal function measured using serum creatinine. (E) Quantification of glomerular crescents. (F) Representative photomicrographs showing severe fibrocellular crescents with H&E stain. (G) Quantification of glomerular CD68+ cell infiltration. (H) Photomicrographs showing representative immunoperoxidase staining for CD68+ cells. (I) Quantification of myofibroblasts using immunoperoxidase staining for αSMA. (J) Photomicrographs showing representative immunoperoxidase staining for αSMA. *N* = 5/group P2RX7 KO, *n* = 6/group WKY WT. All rats are included at each timepoint for serial measurements of urinary abnormalities. Data are representative of one of two independent experiments. Data are shown as median with IQR. Original magnification of images ×400.

To assess the effect of P2RX7 KO on heterologous and autologous phases of NTN, and progression to fibrosis, animals were maintained for 28 days after disease induction. There was equivalent renal injury in P2RX7 KO and WKY WT animals; all had significant urinary abnormalities with proteinuria (Figure 2A,B; median proteinuria 154.7 and 132.9 mg/day for P2RX7 KO and WKY WT, respectively) and haematuria (Figure 2C; median dipstick haematuria 3+ in both groups). There was no difference in excretory renal function between the groups (Figure 2D). At day 28, all animals had severe histological glomerular abnormalities: 60–70% of glomeruli had fibrocellular or fibrous crescents with few normal glomeruli (Figure 2E,F). Glomerular CD68+ monocyte/macrophage infiltration was low, in keeping with the fibrotic stage of disease, and was similar in both groups (Figure 2G,H). alpha smooth muscle actin (αSMA) staining was used to identify myofibroblasts and fibrous crescents; comparable glomerular and interstitial staining was seen in P2RX7 KO and WKY WT rats (Figure 2I,J). There was no difference in deposited heterologous (rabbit) or autologous (rat) IgG within glomeruli. We assessed IL‐1β production by cells infiltrating nephritic glomeruli at an earlier timepoint (day 4) rather than day 28, as this is onset of maximal cell infiltration [18, 32]. There was no difference in IL‐1β release into supernatants from nephritic glomeruli cultured *ex vivo* between P2RX7 KO and WKY WT animals (median interquartile range [IQR] IL‐1β 128.5 (76.4–235.1) and 90.05 (65.7–195.2) pg/1,000 glomeruli for P2RX7 KO and WKY WT, respectively).

**Figure 3:**
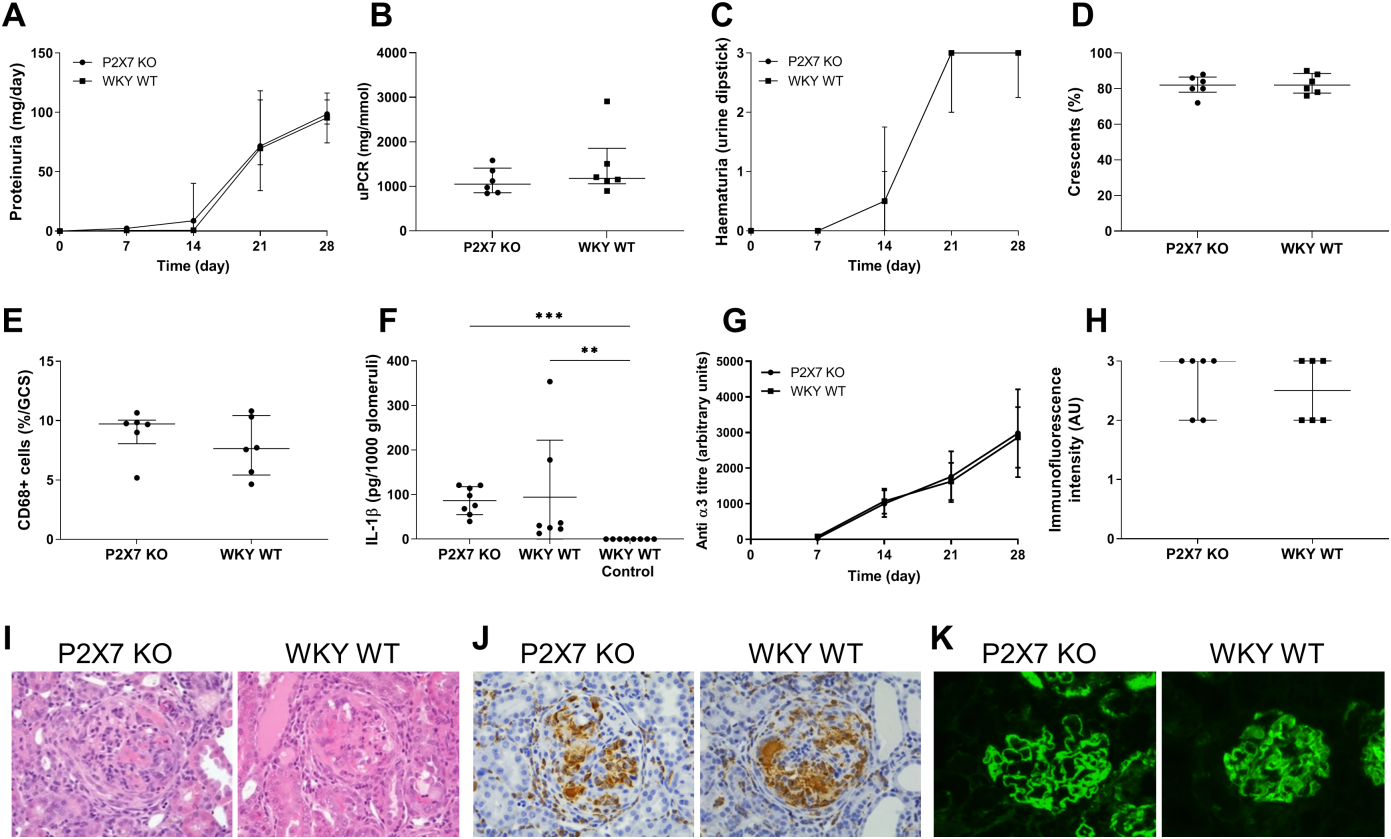
P2RX7 KO rats are not protected from experimental autoimmune glomerulonephritis. (A) Proteinuria during the 28‐day course of EAG. (B) Urine protein: creatinine ratio (uPCR) at day 28 after induction of EAG. (C) Haematuria during the 28‐day course of EAG. (D) Quantification of glomerular crescents. (E) Quantification of glomerular CD68+ cell infiltration. (F) IL‐1β production from nephritic glomeruli at day 28 after disease induction cultured *ex vivo* for 48 h. IL‐1β production expressed per 1,000 glomeruli. (G) Titres of circulating anti‐ α3(IV)NC1 antibody levels throughout the 28‐day course of EAG. (H) Quantification of direct immunofluorescence for deposited anti‐GBM antibodies at day 28 after induction of EAG using antirat IgG FITC. (I) Representative photomicrographs showing severe cellular crescents with H&E stain. (J) Photomicrographs showing representative immunoperoxidase staining for CD68+ cells. (K) Representative photomicrographs of direct immunofluorescence for deposited anti‐GBM antibodies. See also supplementary material, Figure S5. *N* = 6/group from one of two independent experiments. All rats are included at each timepoint for serial measurements of urinary abnormalities. Data are shown as median with IQR and statistical analysis performed using compared Kruskal–Wallis test with Dunn's *post hoc* correction. Original magnification of images ×400.

As P2RX7 may have a role in the development of autoimmunity, or on lung injury, we next investigated the effect of P2RX7 KO in an autoimmune model, EAG [44]. There was no difference in renal injury between P2RX7 KO and WKY WT animals; all had significant proteinuria (Figure 3A,B; median proteinuria 98.5 and 95.5 mg/day for P2RX7 KO and WKY WT, respectively) and haematuria (Figure 3C; median dipstick haematuria 3+ in both groups). Excretory renal function was normal and similar between the groups (supplementary material, Figure S5A). Animals in both groups had severe histological abnormalities; ~80% of glomeruli were affected by cellular crescents with almost no normal glomeruli (Figure 3D,I). Both groups had marked glomerular infiltration of CD68+ cells (Figure 3E,J).

Nephritic glomeruli from animals with EAG cultured *ex vivo* released significant IL‐1β into supernatant compared with glomeruli from control animals and there was no difference between P2RX7 KO and WKY WT animals (Figure 3F; median IL‐1β 86.54, 94.22, and 0 pg/1,000 glomeruli for P2RX7 KO, WKY WT and controls, respectively, *p* = 0.0003).

There was no difference in the severity of lung haemorrhage determined either by visual inspection or by Perls' Prussian blue stain for haemosiderin‐laden macrophages in lung parenchyma (supplementary material, Figure S5B–D; median Perls' stain detection 0.0275 and 0.0255 au for P2RX7 KO and WKY WT, respectively).

Similar results were seen in another autoimmune model, EAV, with no difference in severity of renal or lung injury between P2RX7 KO and WKY WT animals (supplementary material, Figure S6A–L). This model of anti‐MPO vasculitis has distinct pathogenesis, with pauci‐immune glomerulonephritis and lung haemorrhage [34]. In both autoimmune models there was no difference in circulating autoantibodies between P2RX7 KO and WKY WT rats (Figure 3G and supplementary material, Figure S6C). In EAG, all animals had strong linear deposits of IgG along the GBM (Figure 3H,K). In EAV, similar numbers of MPO‐specific B cells were seen in splenocyte preparations from P2RX7 KO and WKY WT (supplementary material, Figure S6D).

### The P2RX7 antagonist A‐438079 prevents the development of renal damage independently of P2RX7

We previously published that A‐438079, an allosteric modulator of P2RX7, decreases the severity of NTN in WKY WT rats [21]. We hypothesised that A‐438079 would be effective in WKY WT but not P2RX7 KO rats if GN is dependent on P2RX7 in WKY WT rats but compensatory P2RX7‐independent pathways mediated GN in the KO.

Rats treated with A‐438079 at 275 μmol/kg or vehicle by twice daily intraperitoneal injection from day 1 (*n* = 4 or 5 per group), were assessed for disease severity at day 7. This dose and route of administration was previously shown to be effective in rat models of nociception, in addition to our previous study in NTN [21, 28].

**Figure 4:**
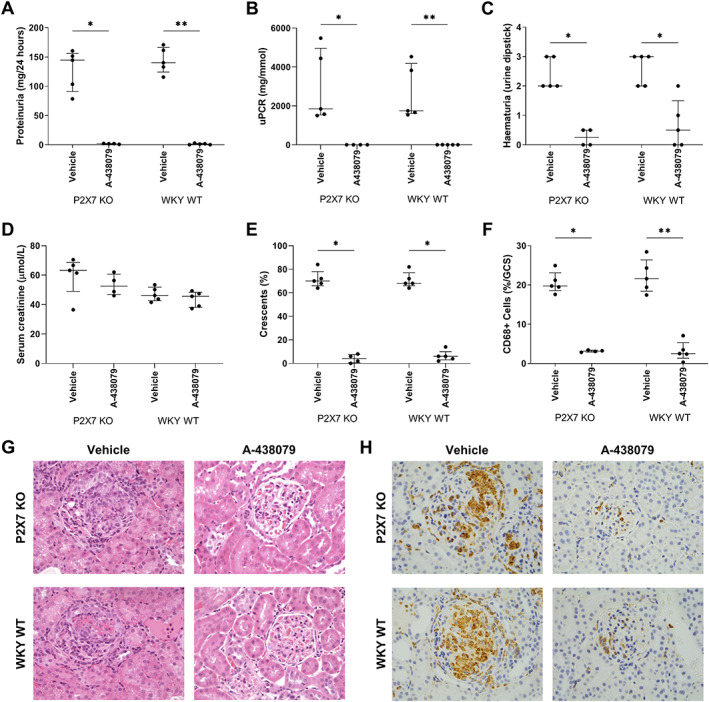
A‐438079 prevents both WKY WT and P2RX7 KO rats from developing nephrotoxic nephritis. (A) Proteinuria, (B) urine protein:creatinine ratio (uPCR) and (C) haematuria following 7 days treatment with A‐438079. (D) Excretory renal function at day 7, measured by creatinine. (E) Quantification of glomerular crescents. (F) Quantification of glomerular CD68+ cell infiltration. (G) Representative photomicrographs of H&E stained kidney tissue showing severe glomerular hypercellularity and fibrinoid necrosis with crescent formation in vehicle treated animals and only mild hypercellularity seen in A‐438079 treated rats. (H) Representative photomicrographs of CD68 immunostaining *N* = 4 or 5 per group. Data are shown as median with IQR. A Mann–Whitney test was used to compare A‐438079‐treated and vehicle‐treated rats. *p < 0.05; **p < 0.01; ***p < 0.001. Original magnification of images ×400.

Contrary to our hypothesis, A‐438079 treatment reduced disease severity in both P2RX7 KO and WKY WT rats. A‐438079 significantly reduced both proteinuria (Figure 4A,B) and haematuria (Figure 4C). Excretory renal function was not impaired and there was no difference between groups (Figure 4D). Histological examination at day 7 showed animals receiving A‐438079 had either normal or mildly hypercellular glomeruli, in contrast to severe abnormalities in vehicle‐treated rats (Figure 4E,G). CD68+ cell glomerular infiltration was significantly reduced by A‐438079 (Figure 4F,H). There was no difference in deposited rabbit IgG or rat IgG between all groups (supplementary material, Figure S7A–D). The effect of the antagonist was not due to a significant effect on circulating leucocytes; white cell count (median (IQR) was 7.1 (4.9–9.9) × 10^9^ and 10.0 (5.7–14.2) × 10^9^ cells/l in antagonist and vehicle‐treated rats, respectively, with no statistically significant difference).

These unexpected results showed that A‐438079 was mediating its protective effects via off‐target, non‐P2RX7‐related pathways. To explore this finding further, a second P2RX7 antagonist, AZ11657312, was used. AZ11657312 is also an allosteric modulator of P2RX7 but is chemically diverse from A‐438079 and likely induces its allosteric effects via a different binding mode. One significant difference between the two inhibitors is the ~50 times greater potency of AZ11657312 against rat compared to human P2RX7 and similar potencies between the two species for A‐438079 [45, 46].

### The P2RX7 antagonist AZ11657312 does not protect rats from developing nephrotoxic nephritis

AZ11657312 was previously shown to be an effective treatment in rat models of diabetes and nociception [29, 45]. Based on previous studies of selectivity for P2RX7, a dose of 60 mg/kg twice daily by oral gavage was used from days 1–7 [45]. AZ11657312 had no effect on proteinuria (supplementary material, Figure S8A) or haematuria (supplementary material, Figure S8B) or excretory renal function (supplementary material, Figure S8C). Glomerular injury was similar between all four groups, with no difference in histological severity, number of CD68+ monocyte/macrophages infiltrating glomeruli (supplementary material, Figure S8D–G), or deposited glomerular rabbit IgG or rat IgG between the groups (supplementary material, Figure S8H–K).

Taken together, these findings, in three experimental models of GN in WKY WT and P2RX7 KO rats, convincingly indicate that P2RX7 is *not* critical in mediating GN or autoimmunity in the rat and that unlike AZ11657312, A‐438079 at the dose used is mediating its effects via off‐target mechanisms. Glomeruli isolated from rats with EAG or NTN, cultured *ex vivo*, produced IL‐1β into the supernatant, and RT‐PCR of renal cortex tissue showed an upregulation of *Il1b* mRNA. This suggests that IL‐1β, generated through P2RX7‐independent pathways, may still play a role in disease. This led us to investigate P2RX7‐dependent and ‐independent mechanisms of IL‐1β production from immune cells.

### Rat bone marrow‐derived macrophages (BMDM) produce cleaved IL‐1β via canonical inflammasome activation

**Figure 5:**
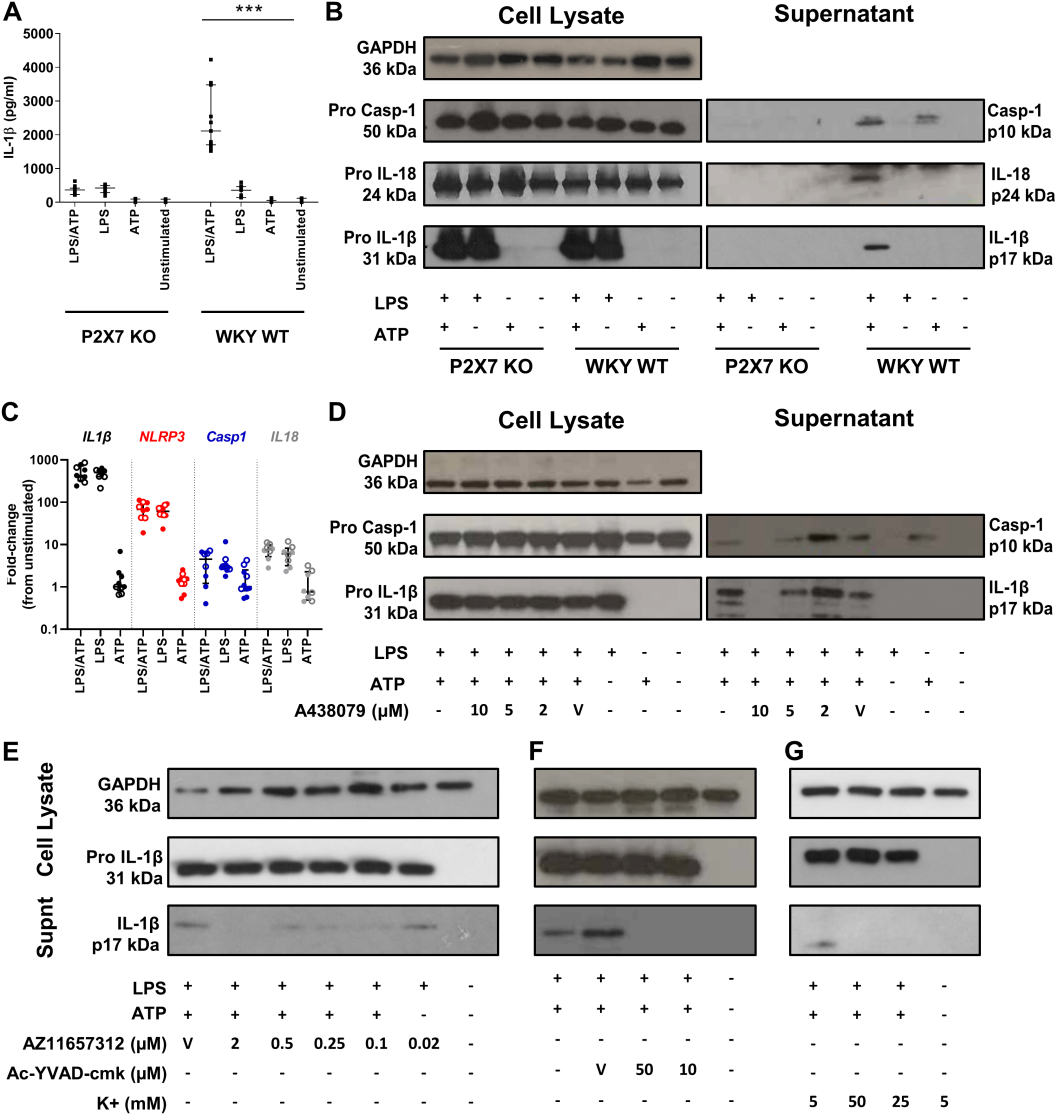
BMDM produce cleaved IL‐1β via canonical inflammasome activation. (A) IL‐1β production by BMDM primed for 4 h with 1 μg/ml of LPS, then stimulated for 30 min with 5 mm ATP. (B) IL‐1β, IL‐18 and caspase‐1 production from BMDM stimulated with LPS and ATP. (C) Gene expression, analysed by RT‐qPCR in P2RX7 KO and WKY WT BMDM stimulated with LPS and ATP as for cytokine measurement. For all gene targets, fold‐change was calculated relative to unstimulated WKY BMDM. Data points for WKY WT BMDM are indicated by closed circles and for P2RX7 KO BMDM by open circles. The effect of (D) A‐438079, (E) AZ11657312, (F) Ac‐YVAD‐Cmk, and (G) Increased extracellular potassium concentration on IL‐1β production from BMDM stimulated as in (A). Western blot images are representative of three biological replicates. Data shown are median, with IQR where appropriate, and compared using a Kruskal–Wallis test with Dunn's *post hoc* correction. ***p < 0.001; **p < 0.01; *p < 0.05. V, vehicle.

As expected, WKY WT BMDM produced significant IL‐1β when primed with LPS and stimulated with ATP, whereas BMDM from P2RX7 KO rats did not. Cells from both rat strains stimulated with LPS alone did not produce IL‐1β (Figure 5A,B). Western blotting of protein precipitated from supernatants confirmed the presence of cleaved IL‐1β, IL‐18, and caspase‐1 in supernatants from WKY WT BMDM stimulated with LPS and ATP (Figure 5B).

The absence of cleaved IL‐1β release from P2RX7 KO BMDM was not due to downregulation of the pro‐form of the cytokine. There was no difference in pro‐IL‐1β levels in cell lysates, and an equivalent increase in *Il1b* mRNA in response to LPS priming in both WKY WT and P2RX7 KO BMDM (Figure 5C).

Two small molecule inhibitors of P2RX7, AZ11657312 and A‐438079 (A‐438079 at a lower dose than was used *in vivo*, previously shown to be selective *in vitro* [28]), and of caspase‐1, ac‐YVAD‐cmk were used to confirm that IL‐1β production by BMDM from the WKY WT rat is dependent on both P2RX7 and activation of caspase‐1 (Figure 5D–F). In keeping with a P2RX7‐dependent mechanism, blocking K^+^ efflux from the cell by increasing the K^+^ concentration of culture media also inhibited IL‐1β release (Figure 5G).

### Bone marrow‐derived dendritic cells (BMDC) produce cleaved IL‐1β independently of canonical inflammasome activation

**Figure 6:**
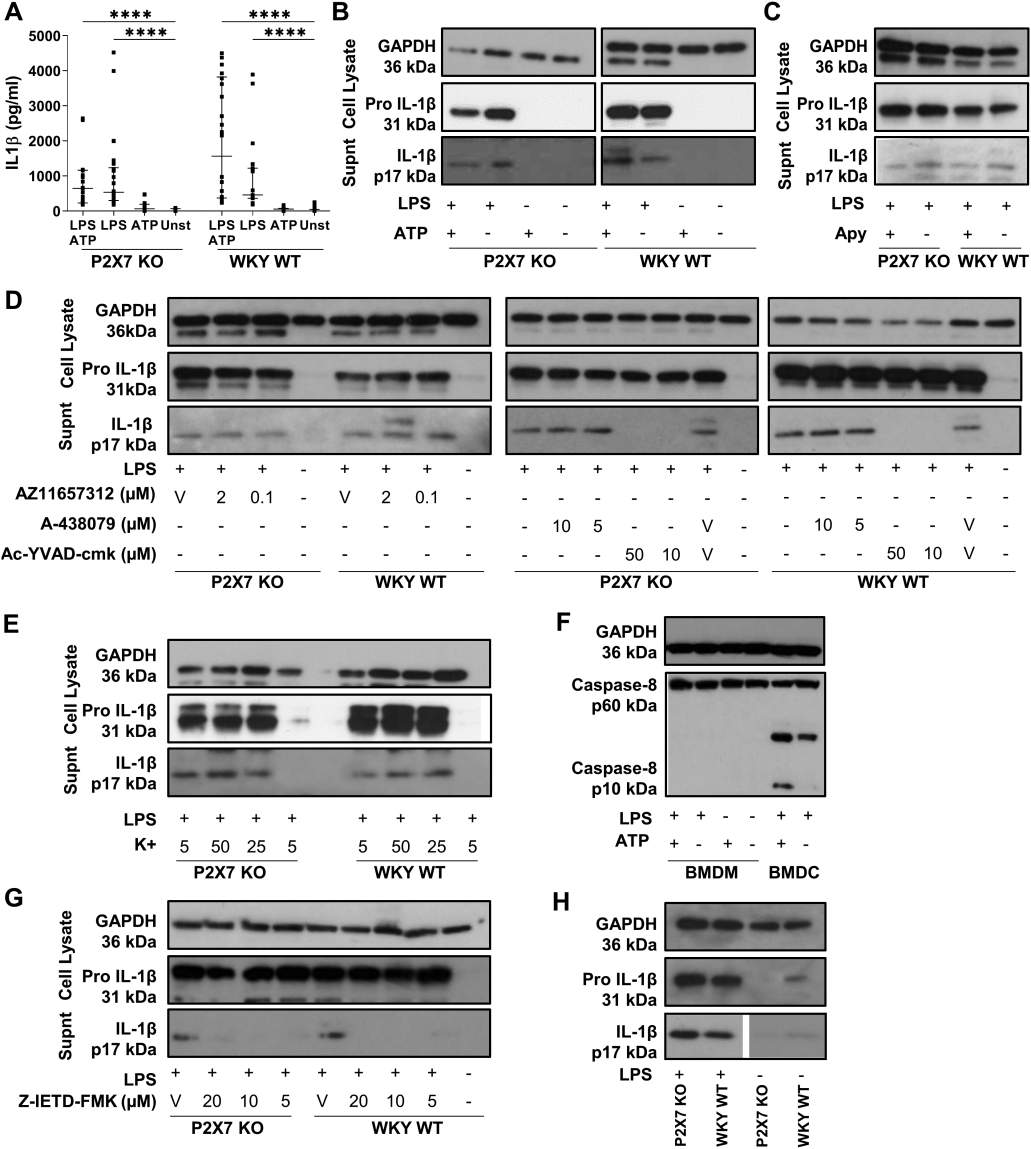
BMDC produce cleaved IL‐1β independently of canonical inflammasome activation. (A) IL‐1β production by BMDC primed for 4 h with 1 μg/ml of LPS then stimulated for 30 min with 5 mm ATP. (B) Western blotting confirming cleaved IL‐1β in supernatant from P2RX7 KO and WKY WT BMDC stimulated with LPS alone. (C) IL‐1β production by BMDC stimulated with LPS in the presence of 10 U/ml apyrase. (D) The effect of A‐438079, AZ11657312, and Ac‐YVAD‐Cmk on LPS‐induced IL‐1β production. (E) The effect of increased culture medium potassium concentration on LPS‐induced IL‐1β production. (F) Levels of activated caspase‐8 (p10 fragment) in cell lysates from BMDC stimulated with LPS (1 μg/ml 4 h), but not BMDM stimulated with either LPS alone or LPS plus ATP 5 mm for 30 min. (G) IL‐1β release in response to LPS (1 μg/ml 4 h) stimulation inhibited by a caspase‐8 inhibitor (Z‐IETD‐FMK). (H) In response to stimulation with LPS alone, there is release of mature IL‐1β into supernatant from both WKY WT and P2RX7 KO monocytes. In cell lysates, pro IL‐1β is upregulated in both WKY WT and P2RX7 KO BMDM in response to LPS stimulation. Data are shown as median with IQR and statistical analysis performed using a Kruskal–Wallis test with Dunn's *post hoc* correction. ***p < 0.001; **p < 0.01; *p < 0.05. V, vehicle.

In contrast to BMDM, significant IL‐1β release was seen following stimulation with LPS alone from both P2RX7 KO and WKY WT BMDC (Figure 6A,B).

ATP was not detected in supernatants from BMDC stimulated with LPS, showing that IL‐1β release in response to LPS alone was not due to an autocrine release of ATP acting at P2RX7 or other receptors. Culture with apyrase to hydrolyse extracellular ATP had no effect on IL‐1β release in response to LPS (Figure 6C). Use of AZ11657312 and A‐438079 confirmed that IL‐1β production was via a P2RX7‐independent pathway; neither compound had an effect on LPS‐induced production of IL‐1β (Figure 6D).

IL‐1β release from BMDC was dependent on caspase‐1, despite being independent of P2RX7; Ac‐YVAD‐cmk completely inhibited IL‐1β production (Figure 6D). This pathway was also identified to be independent of K+ efflux from the cell, suggesting that it is not dependent on canonical or noncanonical inflammasome activation (Figure 6E). We investigated capsase‐8 activation in this pathway, showing active caspase‐8 in BMDC stimulated with LPS, but not BMDM stimulated with LPS +/− ATP (Figure 6F). Use of a caspase‐8 inhibitor, Z‐IETD‐FMK, inhibited LPS‐mediated IL‐1β from both WKY WT and P2RX7 KO BMDC (Figure 6G).

Monocytes from both P2RX7 KO and WKY WT rats could also secrete cleaved IL‐1β in response to stimulation with LPS alone, and it is likely that rat monocytes utilise the same pathways as demonstrated in BMDC (Figure 6H). Due to the limited number of monocytes that it is possible to isolate from rats, the mechanistic studies performed using BMDC were not replicated.

Our results identifying that dendritic cells (and monocytes), but not macrophages, could produce IL‐1β by P2RX7‐independent pathways suggest that these cells may play a role in GN pathogenesis. We used IHC to phenotype infiltrating CD68+ renal leucocytes in EAG. Glomerular cells were predominantly CD43+, suggesting that they represent CD43^hi^ nonclassical monocytes (supplementary material, Figure S9) [37]. Conversely, MHC class II+ cells (dendritic cells) were predominantly peri‐glomerular and tubulo‐interstitial (supplementary material, Figure S9), consistent with other studies suggesting that intravascular CD68+ monocytes (rather than tissue macrophages) are the predominant intraglomerular cell‐mediating injury, and dendritic cells are predominantly found in tubulo‐interstitial infiltrates and adjacent to inflamed glomeruli [37, 47, 48, 49, 50].

## Discussion

We have developed a novel global P2RX7 KO rat with no detectable P2RX7 protein; it is unclear if the double band observed in the brain represents P2RX7 or not. There was no evidence of P2RX7 function in bone marrow‐derived cells, and a bone phenotype, in keeping with the known role for P2RX7 in bone homeostasis [40, 51, 52].

Given the upregulation of P2RX7 in renal cortex from animals with experimental GN, it was surprising that the KO rat was not protected from *in vivo* models of GN and vasculitis. Previous work undertaken in our laboratory showed that P2RX7 KO mice were protected from NTN [21]. There are several reasons why mice could be protected from disease, whereas rats are not. Most importantly, the strain of P2RX7 KO mice used in the earlier study was the GSK P2X7 KO, which has persistence of the P2RX7K splice variant that shows exaggerated responses to ATP [53]. There are also differences in P2RX7 between rats and mice, and although there is 85% P2X7 sequence homology, BzATP is 10‐times more potent against rat (and human) P2RX7 than mouse [46]. There are also several key differences between NTN in mice and models of GN and vasculitis in rat that may account for these findings: mice immunised with nephrotoxic serum alone develop mild disease and require preimmunisation with sheep IgG or co‐administration of LPS is required to increase severity [54]; disease in mice is also milder and more variable, and has a more thrombotic phenotype than that seen in rats (or humans), suggesting that varied mechanisms may be contributing in each species.

We confirmed our previous finding that A‐438079 prevented the development of NTN in rats. In the present study, it was extremely effective in both WKY WT and P2RX7 KO rats, leading us to conclude that it is mediating its actions via ‘off‐target’ effects at the dose used in published *in vivo* studies. We emphasise that our experiments using A‐438079 *in vitro* utilised a lower dose, reportedly highly selective for P2RX7 in characterisation studies [28, 55]. However, the dose used in our *in vivo* experiment (and many other published studies) is nearly 30‐times higher than used for published pharmacokinetic studies [28, 56]. In our previous study 100 μmol/kg A‐438079, which may be more selective for P2RX7, was not effective (and therefore this dose was not repeated here) [21]. It is interesting that this antagonist proved so effective in preventing NTN; investigation of the mechanism(s) by which it is working *in vivo* warrants exploration in future studies and may lead to the identification of novel therapeutic targets.

A second antagonist, AZ11657312, was administered at 60 mg/kg, a dose reported to be effective in rat models, such as the streptococcal cell wall model of rheumatoid arthritis [30, 31]. This compound was also effective in reversing angiotensin II‐mediated renal vasoconstriction and vascular inflammation in rats when used intravenously [57]. AZ11657312 had no effect on GN in either WKY WT or P2RX7 KO rats, suggesting that it may be a more selective antagonist for P2RX7 than is A‐438079. In keeping with this, when used for *in vitro* studies AZ11657312 inhibited IL‐1β release from BMDM, but not BMDC.

Given that P2RX7 is not essential for the development of GN and vasculitis in rats, this raises questions concerning the importance of IL‐1β. Our results implicate IL‐1β in the pathogenesis of GN; there was upregulation of *Il1b* mRNA in the kidney cortex of animals during disease and nephritic glomeruli cultured *ex vivo* produced IL‐1β. Other studies have also demonstrated a role for IL‐1β in experimental GN, and an IL‐1 antagonist has been used to treat NTN in rats [19, 22, 24, 58]. The amount of IL‐1β in glomerular supernatant following *ex vivo* culture was similar for P2RX7 KO and WKY WT glomeruli, suggesting cells producing IL‐1β by P2RX7‐independent mechanisms may play a more important role in disease than those dependent on P2RX7.

*In vitro*, we showed that IL‐1β can be produced by BMDC independently of P2RX7, ATP, and K^+^ efflux from the cell but dependent on caspase‐1 and ‐8. It is likely that rat monocytes can utilise similar pathways. This mechanism may be in keeping with that of ‘alternative’ inflammasome activation described by Gaidt *et al* to be utilised by human but not mouse monocytes, although further studies are needed to address this fully (supplementary material, Figure S1) [9]. In addition to differences in P2RX7 and mechanisms of disease, species differences in inflammasome activation may also explain why P2RX7 KO mice used in the earlier study were protected from GN, whereas P2RX7 KO rats are not.

In NTN and EAG, there is a significant influx of CD68+ cells into the glomerulus during disease and these cells are likely to be the source of IL‐1β when nephritic glomeruli are cultured *ex vivo* [59]. Recent studies using intravital microscopy to examine glomerular leucocytes in NTN have described a role for monocytes rather than tissue macrophages in mediating glomerular inflammation in both rats and mice [37, 49, 60]. In keeping with this, we showed that the CD68+ glomerular infiltrate in EAG is predominantly of CD43+ cells that are likely to be nonclassical monocytes. Thus, infiltrating glomerular monocytes and peri‐glomerular dendritic cells, capable of ATP/P2RX7‐independent IL‐1β generation, may have an important role in propagating disease in these models.

In summary, the findings presented here characterise a novel P2RX7 KO rat and show that P2RX7 is not essential for the development of GN in rodent models. LPS signalling, independent of P2RX7, can lead to production of cleaved IL‐1β in rat BMDC and monocytes, potentially via ‘alternative inflammasome’ pathways. Species and cellular differences in mechanisms of inflammasome activation may, at least in part, explain why—despite several promising preclinical studies in mice—P2RX7 antagonists have not successfully translated into therapeutic agents for human disease. Moreover, for studying inflammasome activation *in vivo*, rats may be more representative of the relevant human pathways. Advances in rat genetic tools over recent years make the species an increasingly attractive alternative to mice for some studies. We contend that the P2RX7 KO rat described here provides an opportunity to study models of inflammation and autoimmunity with greater relevance to human disease than using KO mice.

## Author contributions statement

MP, JB, HTC, RU, CDP, TJA, and FWKT were responsible for conceptualisation. MP, SMc, TTS, AGD, KW, IO, TJA, and FWKT were responsible for methodology. MP, SMc, TTS, AGD, and IO were responsible for investigation. KW, RU, TJA, and FWKT were responsible for resources. MP was responsible for writing of the original draft. RU, CDP, TJA, and FWKT were responsible for writing, review and editing. RU, CDP, and FWKT were responsible for supervision. MP, JB, HTC, TJA, CDP, and FWKT were responsible for funding.

## Supporting information


Supplementary materials and methods
**Figure S1.** Pathways leading to IL‐1β production
**Figure S2.** Characterisation of a novel P2RX7 knockout rat
**Figure S3.** P2RX7 knockout rats do not develop a spontaneous renal phenotype
**Figure S4.** Upregulation of P2RX7, NLRP3 and IL‐1β in rat models of glomerulonephritis
**Figure S5.** P2RX7 KO rats are not protected from lung injury in experimental autoimmune glomerulonephritis
**Figure S6.** P2RX7 KO rats are not protected from renal or lung injury in experimental autoimmune vasculitis
**Figure S7.** A438079 has no effect on deposited glomerular IgG in nephrotoxic nephritis
**Figure S8.** AZ11657312 does not prevent rats from developing nephrotoxic nephritis
**Figure S9.** IHC of renal tissue for monocyte/DC markers
**Figure S10.** Phenotype of BMDC and BMDM
**Table S1.** Primer pairs for RT‐qPCR and standard PCR for sequencing
