# Supplementary material for: Glomerulonephritis and autoimmune vasculitis are independent of P2RX7 but may depend on alternative inflammasome pathways
Source: J Pathol. 2022 May 2;257(3):300–13. doi: 10.1002/path.5890 (PMC9322550; doi:10.1002/path.5890)
Supplement: Supplementary file 1 — Supplementary materials and methods Figure S1. Pathways leading to IL‐1β production Figure S2. Characterisation of a novel P2RX7 knockout rat Figure S3. P2RX7 knockout rats do not develop a spontaneous renal phenotype Figure S4. Upregulation of P2RX7, NLRP3 and IL‐1β in rat models of glomerulonephritis Figure S5. P2RX7 KO rats are not protected from lung injury in experimental autoimmune glomerulonephritis Figure S6. P2RX7 KO rats are not protected from renal or lung injury in experimental autoimmune vasculitis Figure S7. A438079 has no effect on deposited glomerular IgG in nephrotoxic nephritis Figure S8. AZ11657312 does not prevent rats from developing nephrotoxic nephritis Figure S9. IHC of renal tissue for monocyte/DC markers Figure S10. Phenotype of BMDC and BMDM Table S1. Primer pairs for RT‐qPCR and standard PCR for sequencing [file PATH-257-300-s001.docx]

**Glomerulonephritis and autoimmune vasculitis are independent of P2RX7 but may depend on alternative inflammasome pathways**

M Prendecki *et al. J Pathol* DOI: 10.1002/path.5890

**Supplementary materials and methods**

**Supplementary Figures S1–S10**

**Supplementary Table S1**

**Supplementary materials and methods**

Reference numbers refer to the main text list

**Study Approvals**

All animal procedures were carried out in accordance with the regulations of the UK Animals (Scientific Procedures) Act (1986). Animal experiments were carried out under UK Home Office Project Licenses.

**Animal husbandry**

Homozygous P2RX7 KO rats were bred in house, and WKY WT were purchased from Charles River (Saffron Walden, UK). Animals were maintained in a pathogen free facility at the Central Biomedical Services unit, Hammersmith Hospital Campus, Imperial College London, in individually ventilated cages with cage occupancy of 2–5 rats dependent on body weight with free access to water and standard laboratory diet.

**Development of a novel WKY-P2RX7 KO rat strain**

A global P2RX7 KO on a WKY background was created using zinc finger nuclease (ZFN) technology. ZFN mRNA targeting exon 10 of *P2rx7* (Target sequence CATTGGATCCACCCTGTCCTATTTCGGTTTGGTAAGAG) were designed and validated by Sigma-Aldrich (St. Louis, MO, USA). 2.5ng of ZFN mRNA was injected into the pronucleus of single cell WKY WT embryos and embryos transferred into the oviduct of pseudopregnant rats. Offspring were screened using PCR of genomic DNA extracted from ear clippings using primers F AGTGACTGCGAGTTGGTGTG and R ACGAGATGTCTCTCCCCAAA and a 2 base pair insertion in exon 10 confirmed using direct automated fluorescence sequencing. Heterozygous animals carrying the 2 bp insertion were crossed with WT rats and selectively bred to homozygosity, confirmed by sequencing of genomic DNA as above.

**Animal models**

**Nephrotoxic nephritis (NTN)**

Disease was induced in male rats aged 8–10 weeks by injection of a single dose of 0.1 ml of nephrotoxic serum (rabbit anti-rat GBM anti-serum) given by dorsal penile vein injection [32].

**Experimental autoimmune glomerulonephritis (EAG)**

Disease was induced by immunizing 6–8week-old female rats with 100 μg α3(IV)NC1 emulsified in complete Freund’s adjuvant (CFA; Sigma) [33]. Rat α3(IV)NC1 was produced as described previously [33].

**Experimental autoimmune vasculitis (EAV)**

Six–eight-week-old female rats were immunised with purified 1600 μg/kg human myeloperoxidase (MPO; Calbiochem, San Diego, CA, USA) in CFA with additional *Mycobacterium butyricum* (BD, Franklin Lakes, NJ, USA) to a final concentration of 4 mg/ml. Animals also received 500 ng of pertussis toxin (Invitrogen, Waltham, MA, USA) in PBS intraperitoneally on day 0 and day 2 [34].

**Antagonist studies**

For antagonist studies sex-matched littermates were randomised to receive either antagonist or vehicle.

**A-438079 administration**

For in *vivo* studies A-438079 (Sai Life Sciences, Hyderbad, India) was reconstituted in sterile water for injection and a dose of 275 μmol/kg administered twice daily by intraperitoneal injection. Control animals received equivalent injections of vehicle. Animals were treated from day 1–7 after induction of NTN.

**AZ11657312 administration**

For *in vivo* studies AZ11657312 (Astra Zeneca, Cambridge, UK) was reconstituted in sterile water for injection and a dose of 60 mg/kg administered twice daily by oral gavage. Control animals received equivalent gavage of vehicle. Animals were treated from day 1–7 after induction of NTN.

**Assessment of renal and lung injury**

Urine was collected by housing rats overnight in individual metabolism cages with free access to water and standard laboratory diet. Haematuria was quantified by dipstick analysis (Multistix 8 SG; Siemens Healthcare, Erlangen, Germany), and proteinuria was quantified by sulphosalicylic acid method [35]. Blood was collected for serum in plain collection tubes and for cell counts (where applicable) in EDTA tubes. Snap frozen and OCT embedded samples of renal tissue were collected. Renal tissue was fixed overnight in 10% neutral buffered formalin then transferred to 70% ethanol, processed to paraffin blocks and sections stained with periodic acid Schiff (PAS) or haematoxylin and eosin (H&E). Fifty consecutive glomeruli were graded as normal, abnormal (<50% tuft affected by necrosis or crescent), or severely abnormal (>50% tuft affected) by a blinded observer, and results expressed as mean proportion for each animal. For immunohistochemistry primary antibodies were used as indicated including mouse anti‐rat CD68 antibody (ED1, Bio-Rad, Hercules, CA, USA; dilution 1:500), mouse anti-rat CD43 (W3/13, Bio-Rad, dilution 1:100), or mouse anti-rat MHC II (OX-6, Bio-Rad, dilution1:100). This was followed by a biotinylated anti-mouse secondary antibody (Dako, Santa Clara, CA, USA), extravidin-peroxidase (Sigma), and colour developed using 3,3’-diaminobenzidine. The number of positive cells were counted using ImagePro Plus software (Media Cybernetics, Rockville, MD, USA) to assess the percentage staining for 20 consecutive glomeruli; results were expressed as a mean percentage for each animal.

Urine and serum urea and creatinine concentration were analysed in the Clinical Biochemistry Department, Hammersmith Hospital, UK using an AU700 analyser (Olympus). Full blood counts were analysed in the Haematology Department, Hammersmith Hospital using an Alinity HSQ analyser (Abbott, Chicago, IL, USA).

In EAG and EAV severity of lung injury was graded at time of sacrifice after opening the chest cavity but prior to dissection of the lungs using a semi-quantitative scoring system. Lungs were graded as; 0 points- normal macroscopic appearance, 1 point- less than 10 petechiae, 2 points- 10-20 petechiae and 3 points if greater than 20 petechiae were seen.

Lung tissue was collected, paraffin embedded and sectioned as for kidney tissue and Perls’ Prussian blue staining carried out. Haemosiderin laden macrophages were counted using ImagePro software by measuring the proportion of blue-stained cells in 5 high power fields and expressed as mean percentage positive staining for each animal.

**Assessment of autoantibody response**

Circulating α3(IV)NC1 or MPO antibodies were assayed in serum by direct ELISA using pooled sera from historic experiments as a standard curve. ELISA plates were coated with hMPO or α3(IV)NC1 and blocked with 3% BSA in PBS. Rat sera were diluted in PBS and an ALP- conjugated goat anti-rat IgG (1:1000, Sigma) used as secondary. Plates were developed with p-nitrophenyl phosphate solution (Sigma).

The presence of deposited rat IgG (all models) and rabbit IgG (NTN only) was assessed using FITC-labelled antibodies for direct immunofluorescence of frozen kidney sections. Immunofluorescence was assessed by examining 20 glomeruli using a fluorescence microscope and scoring each as 0–3+ with results expressed as mean per animal.

**B-cell ELIspot**

Splenocytes were isolated from rats with EAV 42 days after induction of disease and 500,000 cells/well incubated in ELIspot plates (Multiscreen HTS 96-well filter plates; EMD Millipore, Burlington, MA, USA) which had been coated with 50 μg/ml hMPO. Plates were incubated for 48 h at 37 °C in 5% CO_2_ without moving and developed using a biotinylated rabbit anti-rat immunoglobulin antibody (1:250, Dako), extravadin-ALP conjugate (1:1000, Sigma) and 5-bromo-4-chloro-3-indolyl phosphate/nitro blue tetrazolium (BCIP-NBT) solution (Sigma) Plates were dried and spots per well quantified using an ELISpot plate reader and software (ELISpot 4.0, Autoimmun Diagnostika, Strassberg, Germany).

**Culture of nephritic glomeruli *ex vivo***

Glomeruli were isolated from animals with NTN, EAG and controls by differential sieving of whole-kidney tissue as previously described [36]. Glomeruli were cultured in RPMI supplemented with 10% FCS, 2 mM L-glutamine, and 2% penicillin and streptomycin stock solution for 48 h at 37 °C in 5% CO_2_ and cytokine levels in supernatant measured by ELISA.

**Western blotting**

Cell and protein tissue lysates were prepared NP40 lysis buffer (Invitrogen) supplemented with protease inhibitor cocktail. For supernatants, acetone protein precipitation using 4 volumes of cold acetone incubated at -20 °C overnight, was used. Protein was quantified using a Pierce BCA protein assay kit (ThermoFisher Scientific, Waltham, MA, USA), separated on NuPage® Novex® Bis-Tris minigels (ThermoFisher) and transferred onto a PVDF membrane (Immobilon-P, 0.45 μm, Millipore). Primary antibodies used for Western blot analysis were GAPDH (1:1000, AF5718, R+D Systems, Minneapolis, MN, USA), P2RX7 004 (1:200, Alomone), IL-1β (1:1000, R+D), IL-18 (1:1000, R+D), Caspase-1 (1:2000, Abcam, Cambridge, UK) and Caspase-8 (1:1000, Cell Signaling Technology, Danvers, MA, USA) followed by HRP-conjugated secondary antibodies (R+D) and chemiluminescent reaction (ECL, GE Healthcare, Chicago, IL, USA). Signals were detected using photographic film and an automatic film processor (SRX-10A, Konica Minolta, Chiyoda City, Tokyo, Japan). Semi-quantification of the bands was carried out by optical densitometry, analysed using the ImageJ digital imaging processing software (National Institutes of Health, Bethesda, MD, USA). The expression of each protein analysed was normalized with GAPDH.

**RT-PCR**

Total RNA was extracted from cells and tissues lysed in TRI reagent (Sigma) using the Direct-zol RNA Miniprep kit (Zymoresearch, Irvine, CA, USA) according to the manufacturers protocol including a DNAse 1 digestion step to minimise contaminating genomic DNA. RNA quantity and purity was assessed using A260:A280 ratio and A260:A230 ratio using a Nanodrop 2000c Spectrophotometer (ThermoFisher). Reverse transcription was performed using 1μg of total RNA per sample with iScript^TM^ reverse transcription supermix (Bio-Rad) following the manufacturer’s protocol. mRNA expression was measured by qPCR using primer pairs as detailed in supplementary material, Table S1 and qPCRBIO SyGreen mix (PCR Biosystems, London, UK). Quantification of target gene compared to housekeeping gene was calculated using the comparative Ct method and where appropriate fold-change calculated using the 2^-ΔΔCT^ method.

End point RT-PCR was carried out using goTaq green mastermix (Promega, Wisconsin, USA) using primer pairs shown in Table S1. Agarose gel electrophoresis of PCR products was performed and the QIAquick PCR purification kit (Qiagen, Valencia, CA) was used to clean up remaining PCR products for sequencing analysis. Purified cDNA was sequenced with relevant primers by the MRC CSC Genomics Core Laboratory (Imperial College, London). Sequencing data was analysed with ApE A plasmid editor software (USA).

**Micro-Computed x-ray Tomographic (µCT) analysis**

The tibiae and femora were isolated from 6- and 12-week old P2RX7 KO and WKY WT rats (n=7-9), fixed in 10% neutral buffered formalin (NBF) for 24 hours and stored in 70% ethanol until scanning. µCT analysis of trabecular and cortical bone parameters was performed on the tibial and femoral metaphysis (SkyScan 1172, Bruker, Belgium). The appearance of the first cartilage bridge was used as a reference point, with an offset of 2mm and 5mm for trabecular and cortical bone, respectively. In all cases the length of bone analysed was 2mm. The µCT scanner was set at 50Kv and 200µA using a 0.5mm Al filter and a resolution of 5µm. Analysis of isolated bones was performed without knowledge of the age or genotype. The images were reconstructed, analysed and visualised using SkyScan NRecon, CTAn and CTVol software. Bone mineral density (BMD) was calibrated and calculated using hydroxyapatite phantoms with a known density.

**Primary cell isolation**

Bone marrow was isolated from the femurs and tibiae of 8–16-week-old male and female WT and KO rats. For bone marrow derived macrophages (BMDM), cells were differentiated using 50 ng/ml of recombinant rat MCSF (Peprotech, Rocky Hill, NJ, USA) in DMEM containing 25mM HEPES, 25% FCS, 2 mM L-glutamine, and 2% penicillin and streptomycin stock solution. Cells were cultured for 7 days at 37 °C in 5% CO_2_ and medium supplemented with additional growth factors at the same concentration at 4 days. At day 7 non-adherent cells were washed away and adherent cells collected by incubating with non-enzymatic cell dissociation buffer (Sigma-Aldrich). Bone marrow derived dendritic cells (BMDC) were differentiated using 10 ng/ml recombinant rat GM-CSF (Peprotech) and 10 ng/ml recombinant rat IL-4 (Peprotech) in RPMI containing 10% FCS, 2 mM L-glutamine, and 2% penicillin and streptomycin stock solution. Cells were cultured for at 37 °C in 5% CO_2_, medium was supplemented with additional growth factors at the same concentration at 4 days and non-adherent cells collected for use at day 7. Single cell suspensions of BMDM and BMDC were prepared and stained with fluorescence-conjugated antibodies; P2RX7-FITC (Alomone), Cd11b/c-PE (OX-42, Biolegend), MHC II-APC (His-19, eBioscienc), CD80-PE (3H5, BioLegend) and CD86-PE (24F, Biolegend). For analysis using flow cytometry, cells were washed and re-suspended in sterile PBS containing 0.5% BSA and 1% paraformaldehyde (PFA) and analysed on a BD Accuri C6 flow cytometer (BD Biosciences) and gating strategy and analysis performed using FlowJo v10 software. BMDC and BMDM were phenotypically distinct, with BMDC expressing higher cell surface levels of MHC class II and the co-stimulatory markers CD80 and CD86 by flow cytometry (supplementary material, **Figure S10**).

Rat whole blood was collected in EDTA tubes by cardiac puncture under terminal isoflurane anaesthesia. Monocytes were isolated using cell sorting using a BD FACSAria II flow cytometer. Red cells were lysed using ammonium chloride buffer (pH 7.6) for 6 min on ice. Leucocytes were then washed and incubated with anti-CD45 (OX-1, V450), anti-CD3 (eBioG4.18, PE), anti-B220 (HIS24, PE), anti-CD161a (3.2.3, PE), granulocyte marker antibody (HIS48, biotin), anti-CD43 (W3/13, AlexaFluor647) at 1:40 dilution and anti-rat CD172a (OX-41, FITC) at a 1:8 dilution followed by incubation with streptavidin-PECy7 (1:300 dilution). Monocytes were gated as Lin^–^/CD45+/CD172a+ and CD43/His48 used to generate a monocyte ‘waterfall’[37]. Cells were sorted into 0.5% BSA, washed and classical and non-classical monocytes pooled for stimulation experiments.

**Stimulation of BMDM and BMDC**

Cells were stimulated with 1 μg/ml LPS (ultrapure LPS from E.Coli0111:B4, Invivogen, San Diego, CA, United States) followed by ATP (Sigma) 5 mM for 30 min. Selected cultures also contained 10 units/ml apyrase (Sigma). For P2RX7 antagonist studies cells were pre-incubated with vehicle (0.01% DMSO), 5 μM or 10 μM A-438079 (Tocris, Bristol, UK) or 20 nM –10 μM AZ 11657312 in vehicle for 30 min. For studies using caspase-1 inhibition cells were pre-incubated with vehicle (0.01% DMSO) or 10 μM, 25 μM or 50 μM Ac-YVAD-cmk (Invivogen) and for caspase-8 inhibition Z-IETD-FMK (Invivogen) at 5 μM, 10 μM or 20 μM was used. For experiments in the presence of excess potassium, KCl was added to media to raise K^+^ levels to the indicated concentration. To quantify secreted cytokines supernatants were analysed using sandwich ELISA kit for IL-1β (R+D) according to the manufacturer’s instructions. Western blotting was carried out as described above.

**Yo-pro-1 uptake**

For analysis of Yo-pro-1 uptake cells were suspended in 1ml NaCl buffer (140 mM NaCl, 2 mM CaCl2, 10 mM HEPES, 25 mM Glucose, 1mM MgCl2) warmed at 37 °C and a final concentration of 10μM Yo-pro-1 Iodine (491/509) (Molecular probes^TM^, Invitrogen, Eugene, OR, USA) was added to cell suspensions 5 min prior to analysis. Baseline fluorescence was measured for 1 minute, 150μM BzATP added and dye uptake recorded over 5 min on a BD Accuri C6 flow cytometer (BD Biosciences).

#


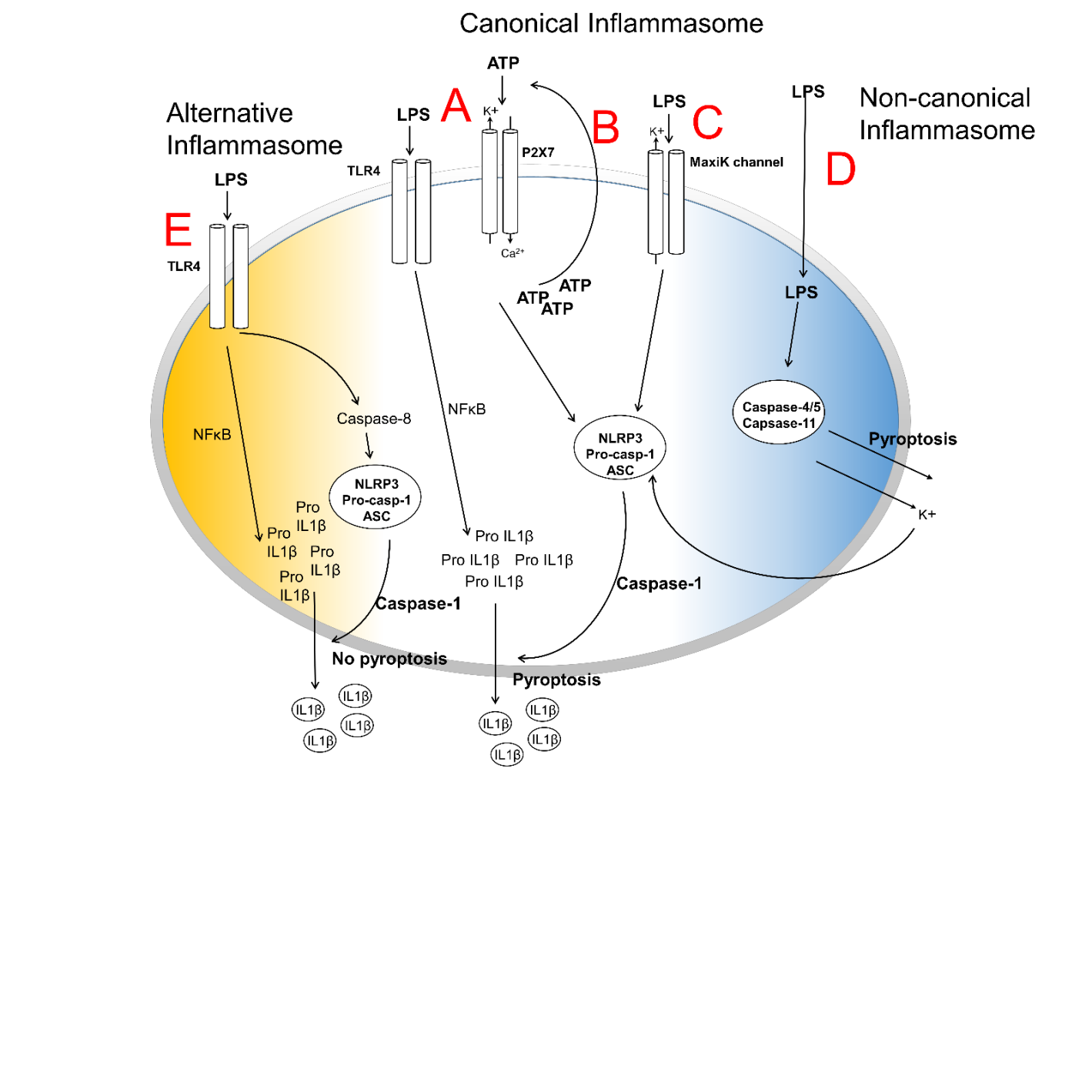


# Figure S1. Pathways leading to IL-1β production

(A) P2RX7 dependent, canonical NLRP3 inflammasome activation is a two-step process. In the first step cells are primed with LPS acts via NFκB pathways leading to accumulation of pro IL-1β within the cytoplasm. ATP acting on P2RX7 then leads to K+ efflux from the cell, activation of the NLRP3 inflammasome and activation of caspase-1 which cleaves IL-1β to its mature form [5]. In human monocytes LPS alone can lead to production of mature IL-1β from cells via a number of postulated mechanisms including:

(B) Canonical inflammasome activation by autocrine release of ATP from cells acting at P2RX7 results in constitutively active caspase-1. When cells are stimulated with LPS resulting in production of pro- IL-1β they are already capable of cleaving it to the active form [11].

(C) Direct activation of MaxiK channels by LPS leading to K+ efflux from the cell and canonical NLRP3 inflammasome activation [10].

(D) Non-canonical inflammasome activation in which in addition to its actions at TLR4, LPS is internalised into the cell and acts at caspase-4/5 (humans) or caspase-11 (rodents) which then leads to pyroptosis, K^+^ efflux from cells and NLRP3 inflammasome activation [12–14].

(E) Alternative inflammasome activation in which LPS acts via TLR4 to induce activation of caspase-8 which in turn activates the NLRP3 inflammasome [9].

Canonical and non-canonical inflammasome activation are dependent on potassium efflux from cells whereas alternative inflammasome pathways are not.


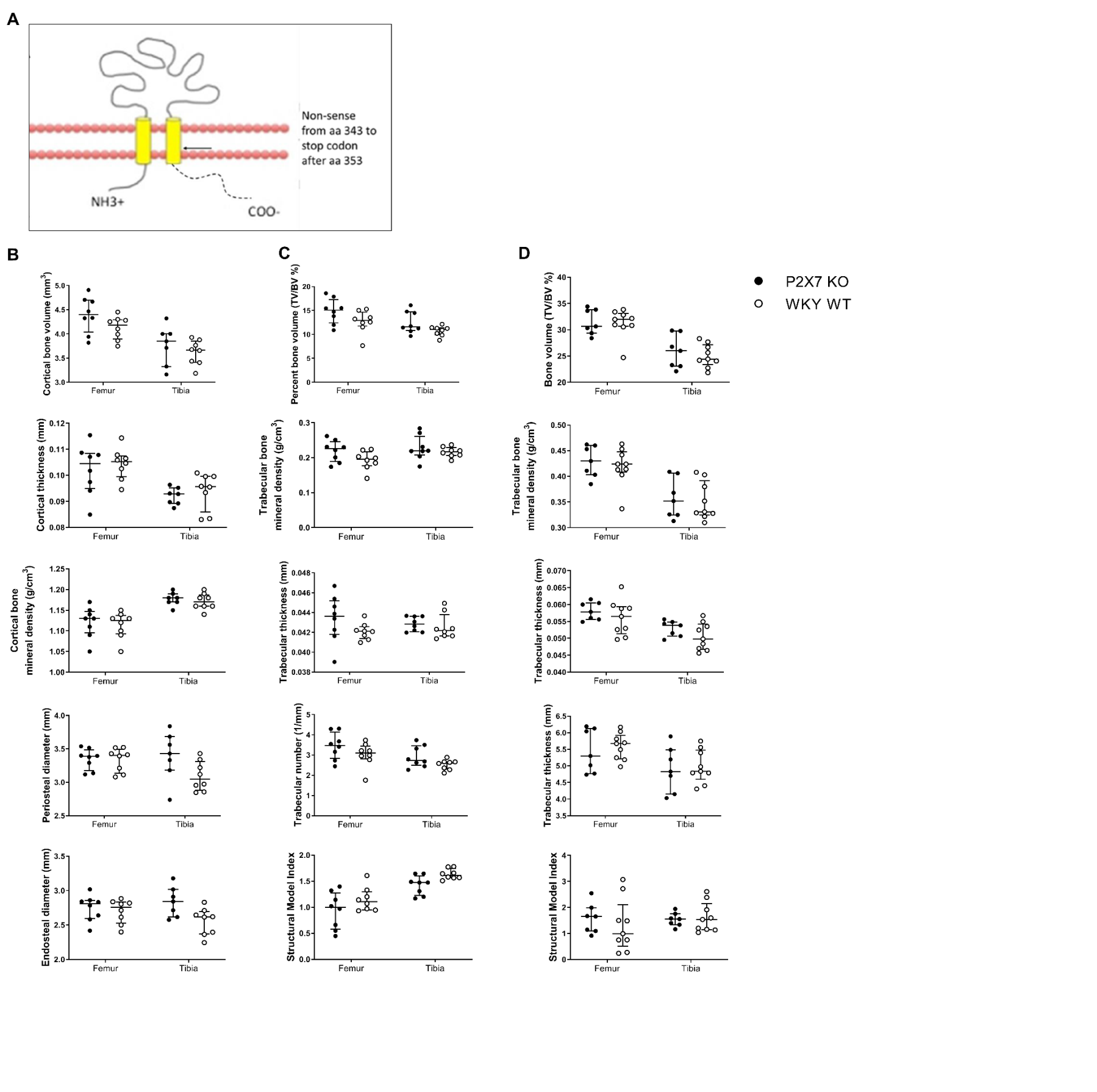


# Figure S2. Characterisation of a novel P2RX7 knockout rat

(A) Schematic diagram of the zinc finger nuclease target site on the structure of P2RX7. Bone phenotyping data showing no difference between P2RX7 KO and age and sex matched WKY WT controls in (B) Cortical bone parameters at 6 weeks, (C) Trabecular bone parameters at 6 weeks and (D) trabecular bone parameters at 12 weeks. Data are shown as mean with SEM.

#
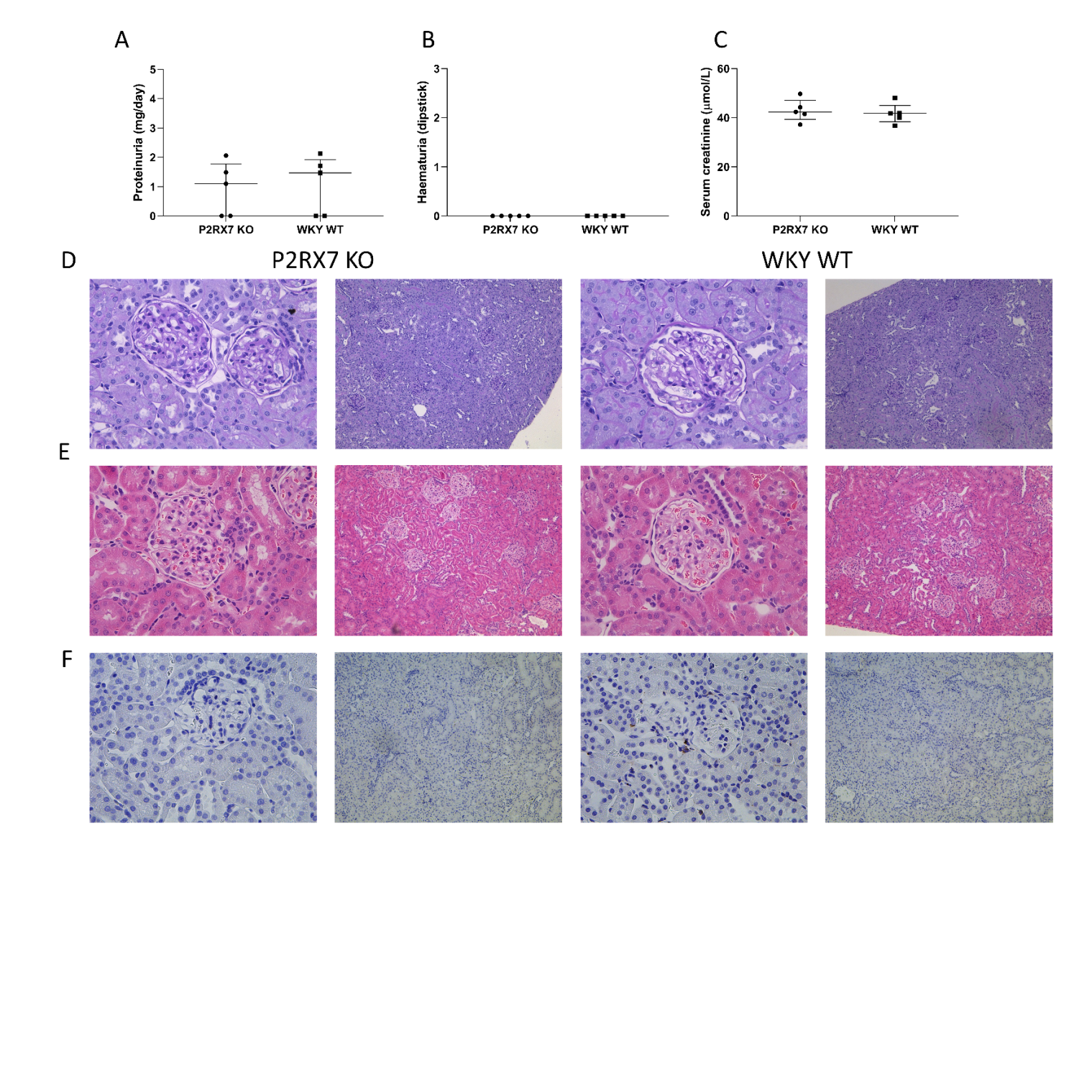


# Figure S3. P2RX7 knockout rats do not develop a spontaneous renal phenotype

(A) Proteinuria (B) Haematuria and (C) Serum creatinine in P2RX7 KO and WKY WT rats. Representative photomicrographs of renal histology at low (x100) and high (x400) power showing no glomerular abnormalities in either P2RX7 KO or WKY WT rats when assessed by (D) PAS stain, (E) H&E stain or (F) IHC for CD68. Data are shown as median with IQR.


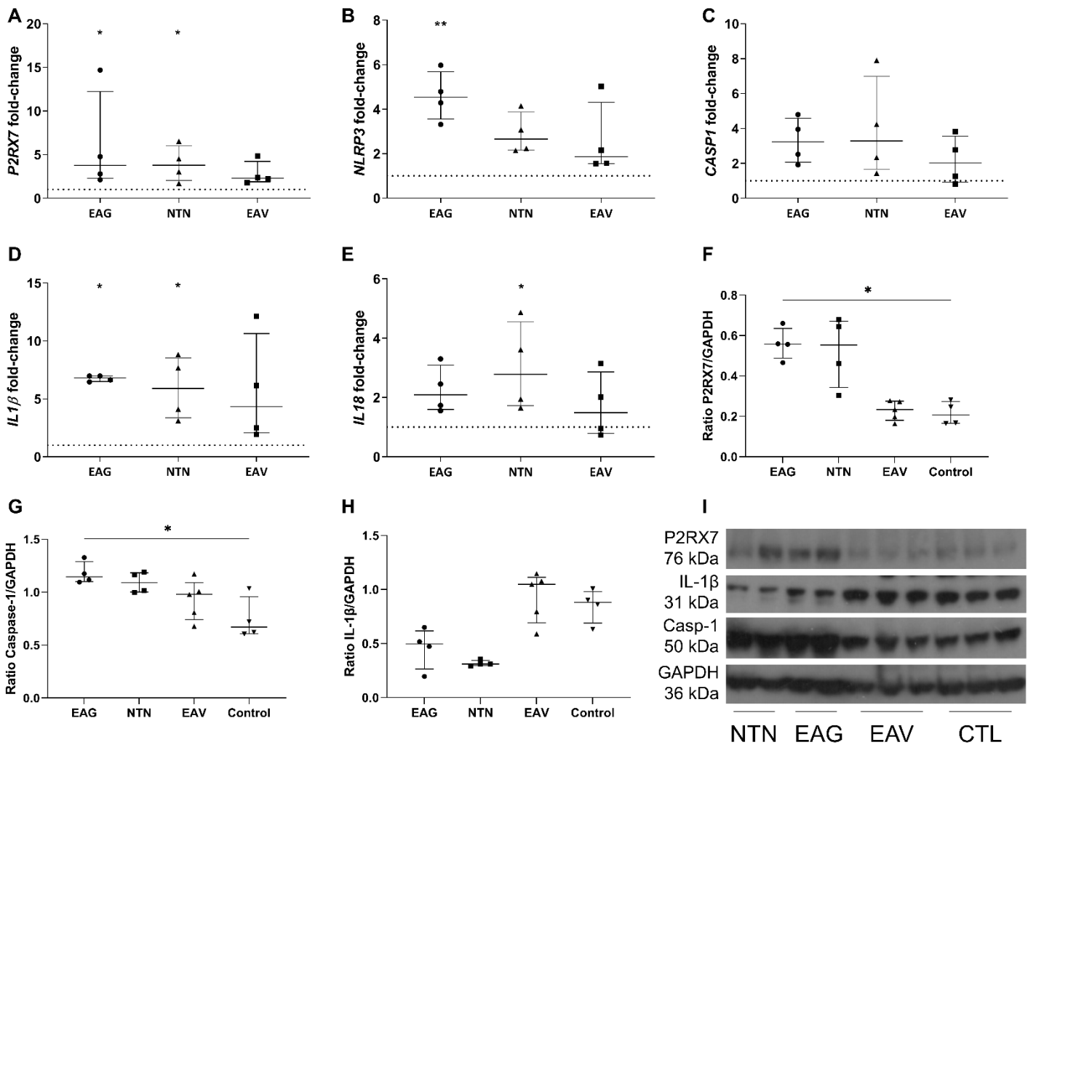


# Figure S4. Up-regulation of P2RX7, NLRP3 and IL-1β in rat models of glomerulonephritis

(A)- (E) RT-qPCR of RNA extracted from renal cortex of WKY WT rats with EAG (day 28), EAV (day 42) or NTN (day 28) showing (A) P2RX7 (B) NLRP3 (C) Caspase-1 (D) IL-1β and (E) IL-18 expression relative to normal rats. (F-I) Quantification of protein expression in renal cortex of WKY WT rats with EAG (day 28), EAV (day 42) or NTN (day 28). (F) P2RX7, (G) Caspase-1 (H) IL-1β compared to loading control (GAPDH). (I) Representative western blot image

Data are shown as median with IQR and statistical analysis performed using Kruskal–Wallis test with Dunn’s *post hoc* correction (comparison to normal/control rats). *p<0.05 **p<0.01


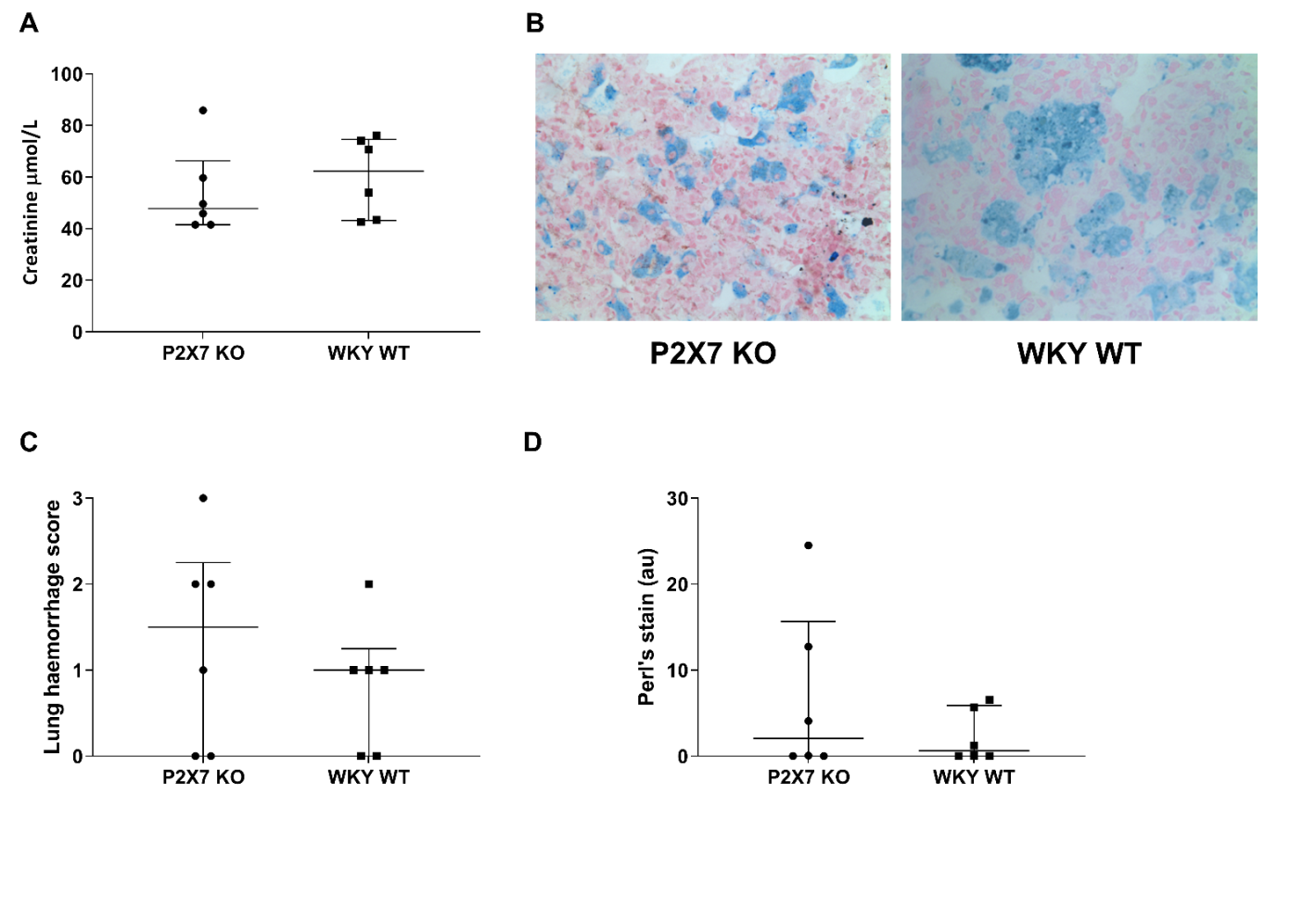


# Figure S5. P2RX7 KO rats are not protected from lung injury in experimental autoimmune glomerulonephritis

(A) Excretory renal function, measured using serum creatinine, (B Representative photomicrographs of lung tissue with Perls’ stain. x200 magnification (C) Severity of lung haemorrhage at day 28 as assessed by visual inspection. (D) Quantification of haemosiderin laden cells in lung tissue using staining of Perls’ Prussian blue per high powered field.

N=6/group, representative of two replicate experiments. Data are shown as median with IQR.

#
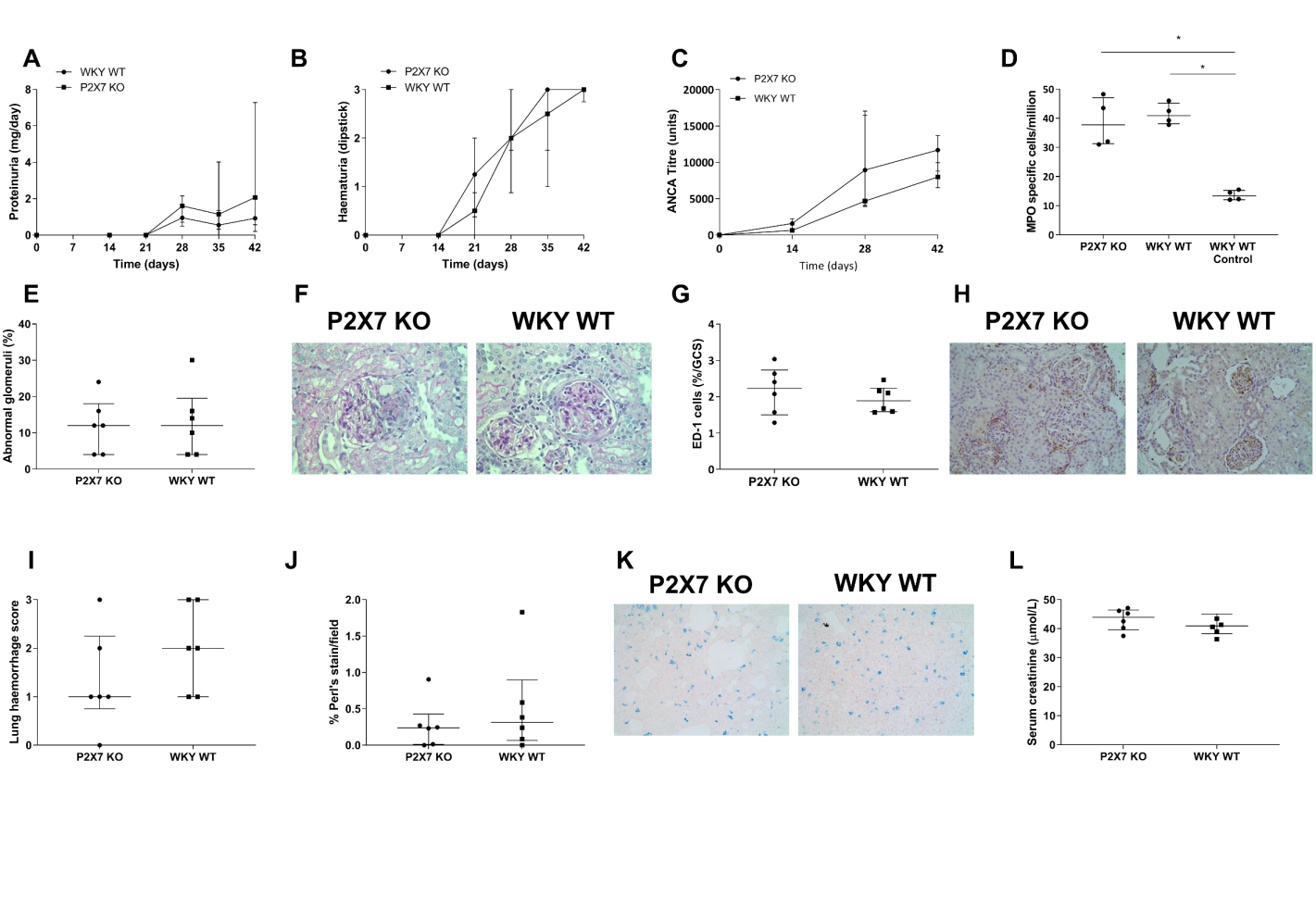


# Figure S6. P2RX7 KO rats are not protected from renal or lung injury in experimental autoimmune vasculitis

(A) Proteinuria and (B) Haematuria during the 42 day course of EAV. (C) Circulating anti-MPO titres (D) MPO-specific B cells identified by B cell ELISpot using splenocytes from rats 42 days after induction of EAV (or immunisation with vehicle) . Results are from 4 biological replicates which were each performed with 4 technical replicates. Each data point is the mean of 4 technical replicates. (E) Quantification of abnormal glomeruli. This is mainly proliferative lesions and segmental necrosis, crescent formation was infrequent. (F) Representative photomicrographs with H+E stain, x400 magnification. (G) Quantification of glomerular monocyte/macrophages using CD68 immunoperoxidase staining. (H) Photomicrographs showing representative immunoperoxidase staining for CD68+ cells, x400 magnification. (I). Severity of lung haemorrhage at day 28 as assessed by visual inspection. (J) Quantification of haemosiderin laden cells in lung tissue using staining of Perls’ Prussian blue per high powered field (K) Representative photomicrographs of lung tissue with Perls’ stain. x200 magnification. (L) Excretory renal function, measured using serum creatinine.

Data are shown as median with interquartile range and statistical analysis performed using a Kruskal–Wallis test with Dunn’s *post hoc* correction was used (comparison to control rats). *p<0.05


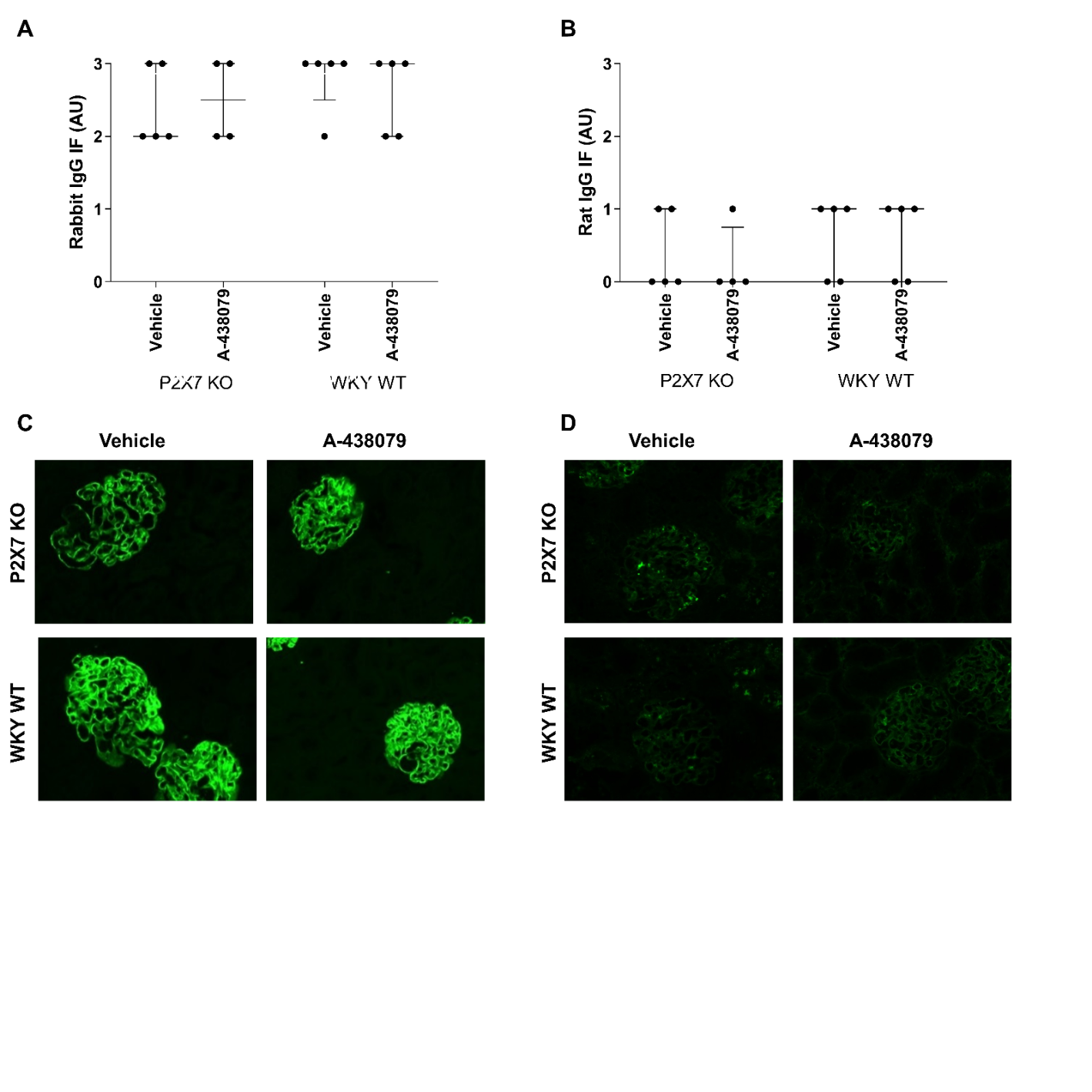


# Figure S7. A438079 has no effect on deposited glomerular IgG in nephrotoxic nephritis

(A). Quantification of direct immunofluorescence for deposited rabbit IgG at day 7 after induction of NTN using anti-rabbit IgG FITC (B) Representative photomicrographs of anti-rabbit IgG FITC. (C). Quantification of direct immunofluorescence for deposited rat IgG at day 7 after induction of NTN using anti-rat IgG FITC (D) Representative photomicrographs of anti-rat IgG FITC.

N=4 or 5/group. Data are shown as median ± IQR. Original magnification of images x400


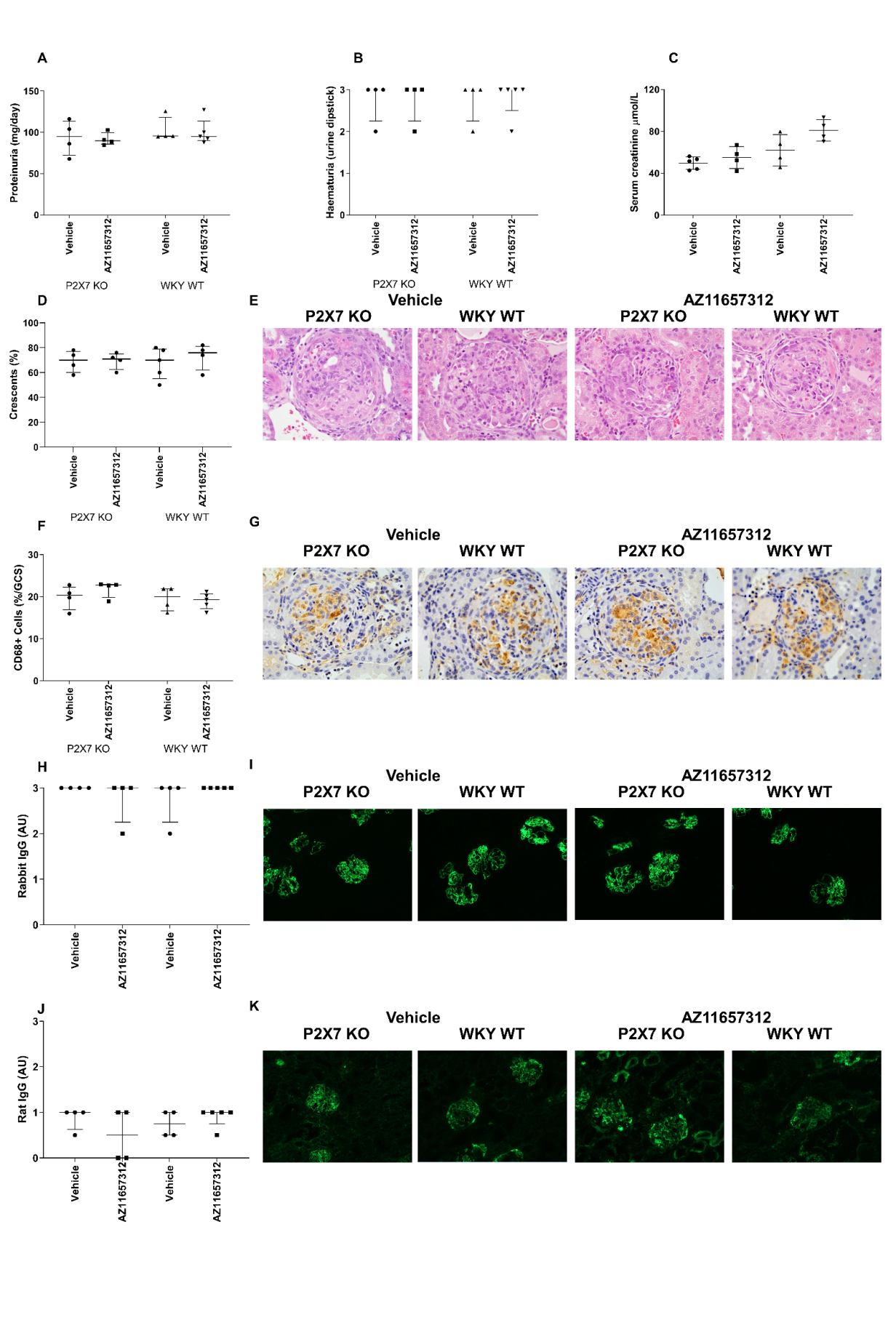


# Figure S8. AZ11657312 does not prevent rats from developing nephrotoxic nephritis

(A) Proteinuria and (B) Haematuria at day 7 after induction of NTN in vehicle treated and AZ11657312 treated animals. (C) Excretory renal function, measured using serum creatinine. (D) Quantification of glomerular crescents. (E) Photomicrographs showing representative glomerular histology with H+E stain. (F) Quantification of glomerular CD68+ cell infiltration. (G) Photomicrographs showing representative immunoperoxidase staining for CD68+ cells. (H) Quantification of direct immunofluorescence for deposited rabbit IgG at day 7 after induction of NTN using anti-rabbit IgG FITC. (I) Representative photomicrographs of anti-rabbit IgG FITC. x400 magnification. (J) Quantification of direct immunofluorescence for deposited rat IgG at day 7 after induction of NTN using anti-rat IgG FITC. (K) Representative photomicrographs of anti-rat IgG FITC. N=4 or 5/group. Data are shown as median ±IQR. Original magnification of images x400 (E–G), x200 (I–K).


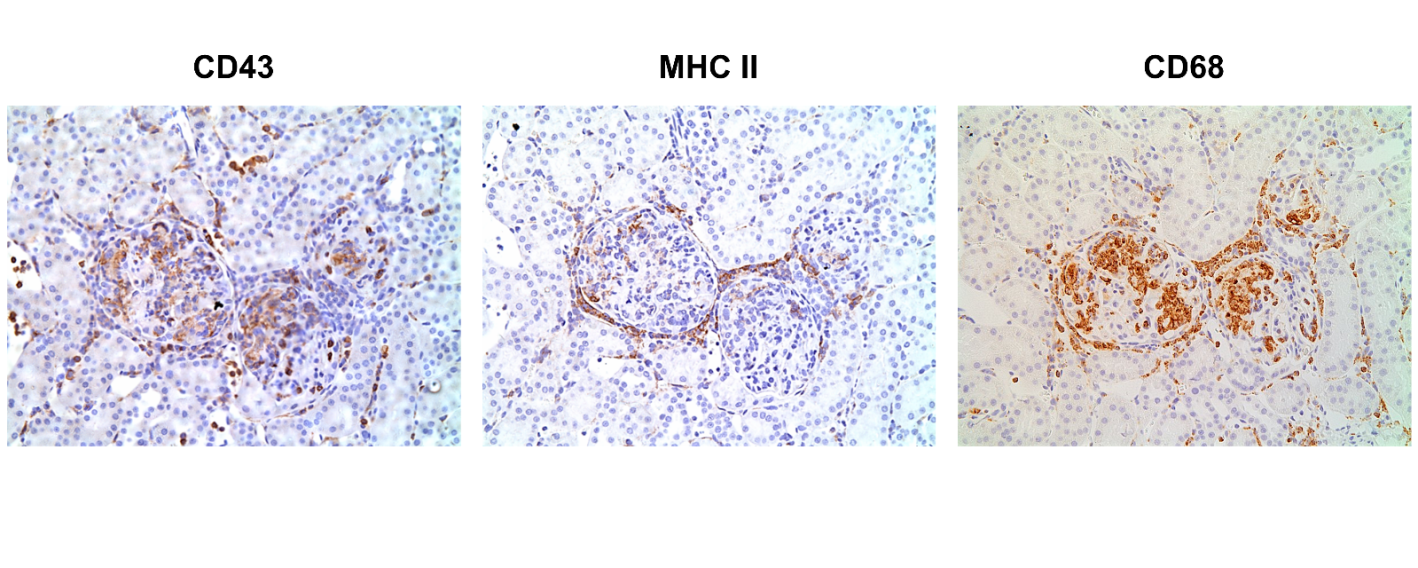


# Figure S9. IHC of renal tissue for monocyte/DC markers

Representative images of immunohistochemistry of serial kidney sections from a rat with EAG for CD43, MHC class 2 and CD68. Original magnification x200 with haematoxylin counterstain.


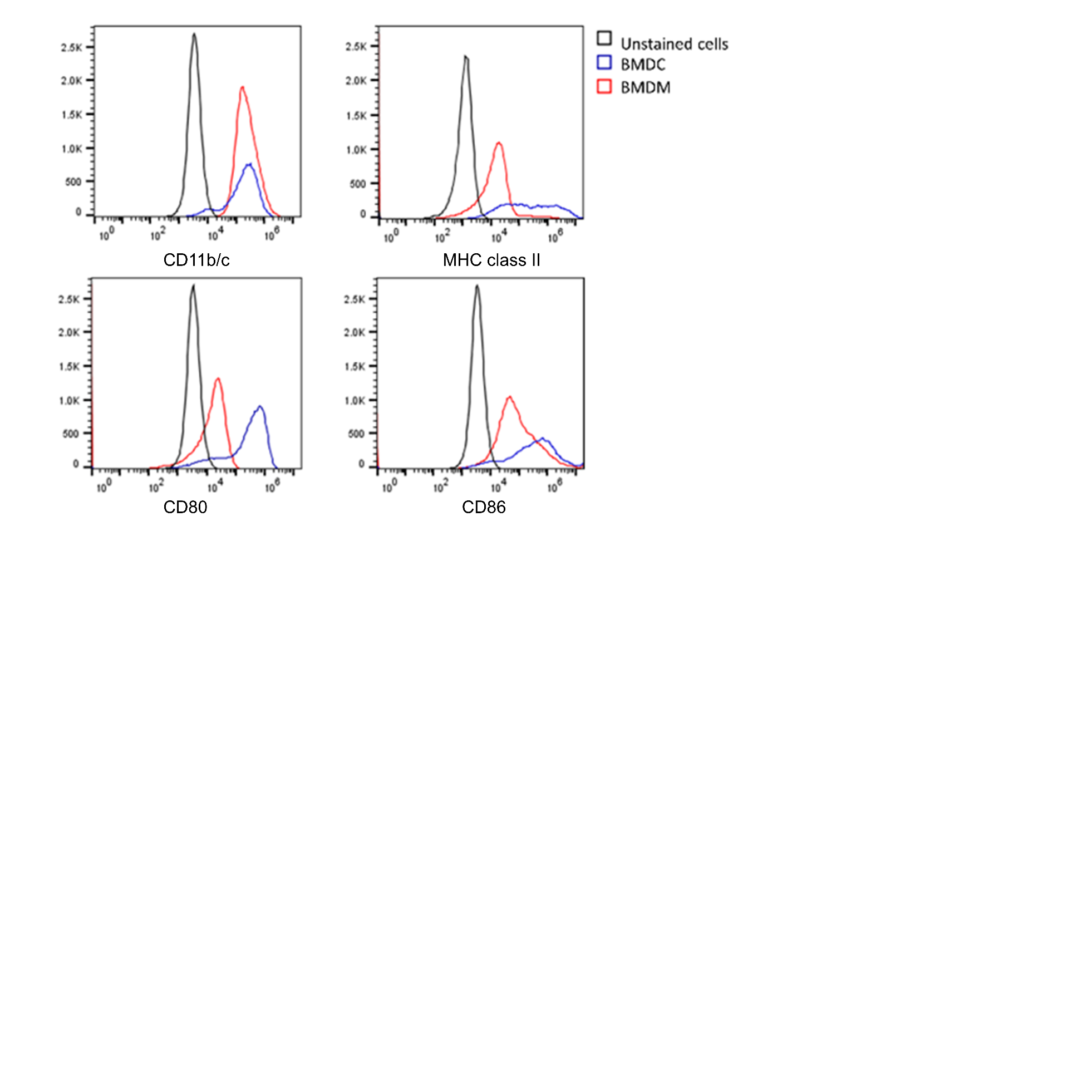


# Figure S10. Phenotype of BMDC and BMDM

BMDC have a different immunophenotype to BMDM with higher cell surface expression of MHC class II, CD80 and CD86. Flow plots are representative of 4 biological replicates. BMDM-Bone marrow derived macrophage BMDC- Bone marrow derived dendritic cell

#

# Table S1. Primer pairs for RT-qPCR and end point RT-PCR for sequencing

| Name | Forward | Reverse |
| --- | --- | --- |
| P2X7A | ACCGTCTTTTCCTACGTTAGCTT | GAGTTCCCCTGCAAAGGGAGG |
| IL-1β | CCTTGTGCAAGTGTCTGAAGC | CAGGTCATTCTCCTCACTGTCG |
| IL-18 | AATGGAGACTTGGAATCAGACC | GGGATTCGTTGGCTGTTCG |
| NLRP3 | CTGCAGAGCCTACAGTTGGG | GTCCTGCTTCCACACCTACC |
| Caspase-1 | TGCCGTGGAGAGAAACAAGG | CCAGGACACATTATCTGGTGTTG |
| PGK1 [61] | ATGCAAAGACTGGCCAAGCTAC | AGCCACAGCCTCAGCATATTTC |
| RT-PCR for sequencing | | |
| P2X7 | CCGTCTTTTCCTACGTTAGCTTT | AGAAGTCCGTCTGGGGTCT |
